# Forming–Aging Pathways and Damage Tolerance in Al–Cu–Mg–Ag Sheets: Mechanisms, Evidence and Implications for Aircraft Skins

**DOI:** 10.3390/ma19204429

**Published:** 2026-10-21

**Authors:** Chenxin Chen, Qingqian Li, Zhaohui Feng, Cheng Chen

**Affiliations:** 1 Beijing Key Laboratory of Intelligent New Materials and Application Demonstration for Civil Aircraft, COMAC Beijing Civil Aircraft Technology Research Center, Beijing 102211, China; 2 Beijing Engineering Research Center of Advanced Aluminum Alloys and Applications, Beijing Institute of Aeronautical Materials, Beijing 100095, China

**Keywords:** Al–Cu–Mg–Ag alloy, sheet forming, Ω precipitation, prestrain, stress aging, fatigue crack growth, damage tolerance, residual stress

## Abstract

In Al–Cu–Mg–Ag sheets considered for aircraft skins, forming and aging alter the state relevant to subsequent fatigue and fracture. Favorable precipitation and tensile responses alone do not establish retention of a specified fracture or fatigue property. This critical narrative review evaluates composition and inherited state, deformation–aging sequence, and separately defined damage endpoints. Documented supplementary searches and evidence mapping support the authors’ engineering and scientific assessment. Chemistry and thermal history jointly control competing precipitates; a smaller Ω volume fraction can coexist with higher yield strength, while resistance to stable tearing can decrease after thermal exposure even when yield strength changes little. Responses to small tensile prestrain, rolling, loading during aging and warm compression depend on the initial state and intervention timing. Tear tests, three-dimensional damage observations, fatigue-life distributions and fatigue-crack-growth measurements reveal distinct roles for particles, boundaries, texture and initiation sites. Cross-over alloys offer bounded comparisons of forming–hardening strategies. The synthesis identifies controls for comparing complete processing routes and locating susceptible regions. Process-retained damage tolerance provides a reporting framework that preserves absolute performance alongside relative change and distinguishes mechanism identification from route optimization. Future work should connect mapped local histories to matched crack endpoints and representative curved structures.

## 1. Introduction and Scope

Aluminum fuselage skins must sustain repeated loading while tolerating manufacturing imperfections and service damage. Their selection consequently involves strength, crack-growth resistance, residual strength, corrosion behavior and manufacturability. Reviews of aircraft aluminum development show that improvements in one property have repeatedly required adjustments elsewhere in this combination [1,2,3]. Integral and locally reinforced panel concepts add further coupling between material conditions and structural geometry [4]. For a formed skin, assessment must therefore concern a material at a defined location after its complete processing history rather than a nominal alloy composition evaluated in an unrelated product form.

Al–Cu–Mg–Ag alloys are attractive in this context because their precipitation behavior permits useful combinations of strength and thermal stability. However, their development history also illustrates why sheet, plate and formed-component results must remain distinguishable. NASA investigations of RX226-T8 and alloy 2139 examined orientation, thickness location, toughness and crack growth in specific plate products [5,6]. These studies provide a material basis for aircraft applications, but they do not demonstrate that every sheet-forming route preserves those properties. The present review concentrates on how forming and aging alter the state from which fatigue and fracture subsequently develop.

An integrated treatment of forming and damage tolerance has clear precedents. Starink and co-workers related microstructure, age formability and fatigue-crack-growth resistance in Li-containing alloys, while the AGEFORM project combined precipitation and creep studies with static and damage-tolerance testing and structural demonstrators [7,8,9]. In the 2006 AGEFORM account, AA2139 appears in four-point-bending and strength–apparent-toughness comparisons; the explicitly matched fuselage-sheet R-curve comparison concerns AA2022. These achievements establish the basis for the present question: which local deformation–aging changes in Ag-bearing sheets explain differences between damage endpoints, and which controls can distinguish them?

Precipitation reviews explain alloy chemistry and hardening sequences; hot-forming and springback reviews organize forming routes and shape control; residual-stress reviews explain manufacturing distortion [10,11,12,13,14]. This review connects these bodies of work through three material-specific comparisons. First, it distinguishes apparently inconsistent Ω responses by the initial state, deformation timing, onset of plasticity and the observable being measured. Second, it relates those descriptors to crack initiation, small-crack growth, long-crack propagation, and tearing, preserving the stages for which a connection has been observed. Third, it converts the resulting distinctions into conditional process controls and complementary material and component comparisons. The contribution is the connection between mechanisms, measured endpoints, and usable controls rather than a claim that coupled alloy–forming–property assessment is new.

Building on earlier integrated alloy–forming–property assessments [7,8,9], process-retained damage tolerance (PRDT) is used here as a reporting framework that links each processing intervention to the local tested state, an explicit reference condition and a separately defined endpoint. Its contribution is to make the conditions of a retention claim traceable, while retaining absolute performance alongside relative change and distinguishing mechanism-identification controls from route optimization. PRDT does not introduce a new material property or scalar damage-tolerance index, nor does it replace fracture-mechanics-based or structural damage-tolerance assessments.

Recent atom probe tomography (APT) studies of small prestrain and new comparisons of deformation order sharpen the link between the state entering deformation and subsequent precipitation. Earlier three-dimensional tearing studies establish complementary observations of localization and void linkage. Bringing these sources together makes it possible to ask which processing changes are supported by a measured damage endpoint and which still require a matched test.

Four objectives guide the review: (O1) identify how compositions and inherited states delimit material comparisons; (O2) distinguish deformation mode, load, and timing when interpreting precipitation and forming responses; (O3) connect local observations to separately defined fracture, crack-growth, and fatigue-life endpoints; and (O4) formulate matched comparisons and bounded transfer criteria from processed coupons to aircraft skins.

## 2. Review Scope, Search and Evidence Synthesis

### 2.1. Review Type, Search and Assessment

This article is a critical narrative review, not a comprehensive systematic review. Documented supplementary searches, targeted source assessment and evidence mapping support a question-led synthesis of forming–aging pathways, microstructures, and damage-related properties in Al–Cu–Mg–Ag alloys; they do not establish exhaustive coverage of the literature. The author team selected the literature, extracted and interpreted the relevant information, and made the scientific judgments used in the synthesis, drawing on engineering experience in aircraft structures and materials processing. No new, unpublished experimental data are presented; quantitative figures reproduce or descriptively summarize results from the cited literature. No preregistered protocol or independent dual screening was used; these are limits of the procedures reported, rather than criteria that alone determine the review type.

The latest recorded discovery batches were executed in September 2026, principally through ScienceDirect. Seven ScienceDirect queries yielded 1003 saved occurrences and 637 distinct Publisher Item Identifiers (PIIs). Of these, 69 matched available full texts: 37 were represented in the accumulated targeted-assessment register and 32 were outside it. Neither an unmatched record nor a record outside that register was automatically excluded. The all-source register contained 110 reports assessed at different depths. Two further reports were assessed specifically for the replacement micrographs; these were additions to the bibliography and not to the archived search and assessment cohorts. The final bibliography contained 126 distinct reports: 82 were retained from the original submission with 44 additions. These counts describe different sets; Methods S1 and Figure S1 describe the executed queries and counting relationships.

Selection for a particular synthesis depended on which question the source could answer. The author team prioritized traceable material identity and processing history; a defined observation or endpoint; interpretable test conditions; and a comparison relevant to the composition, timing, local damage, or structural transfer. Quantitative comparisons required compatible endpoint definitions and sufficient material and test metadata; sources with unresolved decisive conditions were restricted to qualitative use. Reviews and indirect alloy analogs supplied backgrounds or methods, while reports retained for a possible later question were designated as a reserve. Related publications were linked to avoid treating reused baselines as independent evidence. These decisions developed iteratively during the review rather than through a preregistered score. Study conditions, measured endpoints, and limits of comparison were recorded to support the evidence synthesis. Measurements, source-author interpretations, model outputs and the present synthesis are distinguished. NR denotes unreported information, NE refers to information not extracted or unresolved, and NA is an inapplicable field.

Results measured in Al–Cu–Mg–Ag alloys are identified as target-family evidence, with product form and test conditions retained. Results from other aluminum-alloy systems are used as explicitly identified mechanistic or methodological comparisons and not as direct validation of Al–Cu–Mg–Ag sheets. Evidence obtained from target-family plate, extruded, model-crystal or additively manufactured material is also distinguished from evidence obtained from wrought sheet or formed panels.

### 2.2. Defining Endpoints Before Comparing Processes

In this review, damage tolerance refers to the ability of a structure containing specified damage to sustain the required loads while accounting for crack growth under stated service conditions. Fracture resistance, fatigue crack-growth behavior, and structural residual strength provide distinct inputs to its assessment; no individual material test result is used as a synonym for structural damage tolerance. Tensile properties and total fatigue life are discussed separately as strength and durability measures. A tear test, a fatigue-crack-growth test and a residual-strength test probe different stages of damage and different constraint conditions. AA2139 tear measurements and crack-growth experiments on Ag-bearing Al–Cu–Mg materials demonstrate this separation directly [15,16]. Large-crack experiments in thin AA2024-T3 sheets further show why small-scale yielding and geometry assumptions require attention as failure approaches [17]. Table 1 defines the endpoints used here. In particular, a model value with stress-intensity units is not automatically a valid plane-strain toughness measurement, and a fatigue-strength factor at a selected cycle count is not a fatigue-life multiplier. In the welded-sheet comparison, fatigue strength is expressed as maximum stress, σ_max_, at the specified cycle count; it must not be replaced by stress amplitude.

**Table 1:** Endpoint definitions used to prevent incompatible comparisons. The listed properties and test endpoints are not interchangeable measures of damage tolerance; their relevance depends on the specified damage scenario, loading conditions, and structural configuration. This table is a review synthesis and not a replacement for the applicable test-method requirements.

Endpoint	What the Results Support	Comparison Conditions
Yield strength (YS), ultimate tensile strength (UTS) and elongation	Tensile response; screening of strength–ductility balance	Direction, gauge, strain rate, temperature and heat-treatment history
Kahn unit initiation energy (UIE, N/mm)	Geometry-specific work to the defined initiation point	Identical specimen geometry, orientation and integration convention
K_IC_ or an appropriate resistance curve	Fracture resistance subject to the applicable validity and constraint conditions	Thickness, crack geometry, orientation, loading and validity assessment
da/dN versus ΔK	Crack-growth rate under specified cyclic conditions	Load ratio, frequency, environment, crack regime and stress state
Strain-controlled fatigue: N_f_ cycles or 2N_f_ reversals	Cyclic plasticity and strain–life response; reversals are not cycles	Total or plastic strain amplitude, strain ratio, waveform, frequency, geometry and failure convention
J–R-derived Kc at a specified crack extension	Source-defined resistance to stable tearing under monotonic loading, e.g., √(JE) at Δa = 2 mm [18]; not automatically K_IC_	J evaluation, crack extension, width, thickness, orientation and validity
Fatigue life N_f_ and its sample distribution	Total lifetime under specified loading; includes initiation and propagation	Stress definition, load ratio, frequency, environment, geometry, sample size and run-out treatment
Fatigue strength at a specified cycle count	Measured or fitted stress at a stated finite life; σ_max_ in the welded-sheet comparison	Cycle count, maximum stress or stress amplitude, fitting method, censoring and uncertainty
Immersion-corrosion depth; stress-corrosion cracking (SCC); corrosion fatigue	Distinct environmental responses, not interchangeable endpoints	Exposure chemistry and duration, sustained or cyclic load and specimen conditions
Residual strength of a cracked panel	Structural response for a defined damage scenario	Crack configuration, load transfer, curvature, boundary conditions and thickness

## 3. The Material State Inherited by Forming

### 3.1. Ω Precipitation Is an Interfacial and Competitive Process

The initial state for forming comprises more than the solute content. It includes supersaturation, vacancy availability, clusters, precipitate populations, dislocations, grain structure and texture. General treatments of precipitation in aluminum emphasize that these descriptors evolve across different time and length scales and contribute differently to strength [10,19,20,21]. Early work on low-Cu/Mg Al–Cu–Mg–(Ag) alloys has also shown that rapid cluster hardening can precede later precipitate development [22]. This result does not define a universal precipitation sequence for Ω-strengthened alloys. It shows why the intervals between quenching, deformation, and aging can be a material variable.

Ω forms as plates on {111}Al, and its broad interfaces provide a central link between chemistry and stability. Atom probe work identified Mg and Ag segregation associated with Ω interfaces [23]. Atomic-resolution imaging combined with first-principles calculations subsequently showed how interfacial chemistry can lower formation energy and accommodate misfit through changes in atomic occupancy [24]. These findings support an interfacial description of strengthening precipitates rather than treating their nominal bulk formula as a complete state descriptor. For forming, the implication is conditional: any process that changes the available solute, defect-assisted transport, or populations of competing precipitates can alter the route by which Ω distribution develops.

The precursor structure also matters. Yang and co-workers identified a metastable Ω′ plate with a hexagonal structure and demonstrated its in situ evolution into Ω using atomic-resolution microscopy and supporting calculations [25]. A substantial shear component accompanied the proposed precursor transformation. This evidence refines the mechanistic basis for discussing stress and orientation effects, but it does not establish a numerical stress–precipitation law for polycrystalline forming. Likewise, in situ hot-stage transmission electron microscopy (TEM) showed strong interactions between matrix dislocations and growing Ω plates [26]. Together, these observations make the deformation history relevant to both nucleation and subsequent growth while leaving the dominant response dependent on the temperature and starting state.

Thermal stability is similarly conditional. Hutchinson and co-workers associated the strong coarsening resistance of Ω plates with the barrier to ledge nucleation; interfacial segregation alone did not explain the observed temperature dependence [27]. Gable and co-workers connected long-term stability with alloy chemistry and competition from S-phase precipitation [28]. For manufacturing, thermal stability therefore means stability under a specified time–temperature–stress history. Resistance to coarsening in a static exposure cannot, by itself, establish resistance to deformation-assisted evolution or to the loss of fatigue performance during a later thermal cycle.

### 3.2. Chemistry and Composition–Treatment States

Mg modifies Ω precipitation through both clustering and competition for solutes. Quantitative atom probe and TEM studies showed that the number of initial Mg–Ag co-clusters is not the sole determinant of the later Ω population [29]. Work on solute partitioning further related composition-dependent precipitation to the chemistry of the evolving plates [30]. These studies show that the initial cluster population does not uniquely determine the subsequent Ω population or its strengthening contribution. The implication for fracture resistance requires a separately defined damage endpoint. The following composition–treatment comparison provides Kahn UIE alongside YS, while later sections consider direct crack-growth measurements.

**Figure 1:**
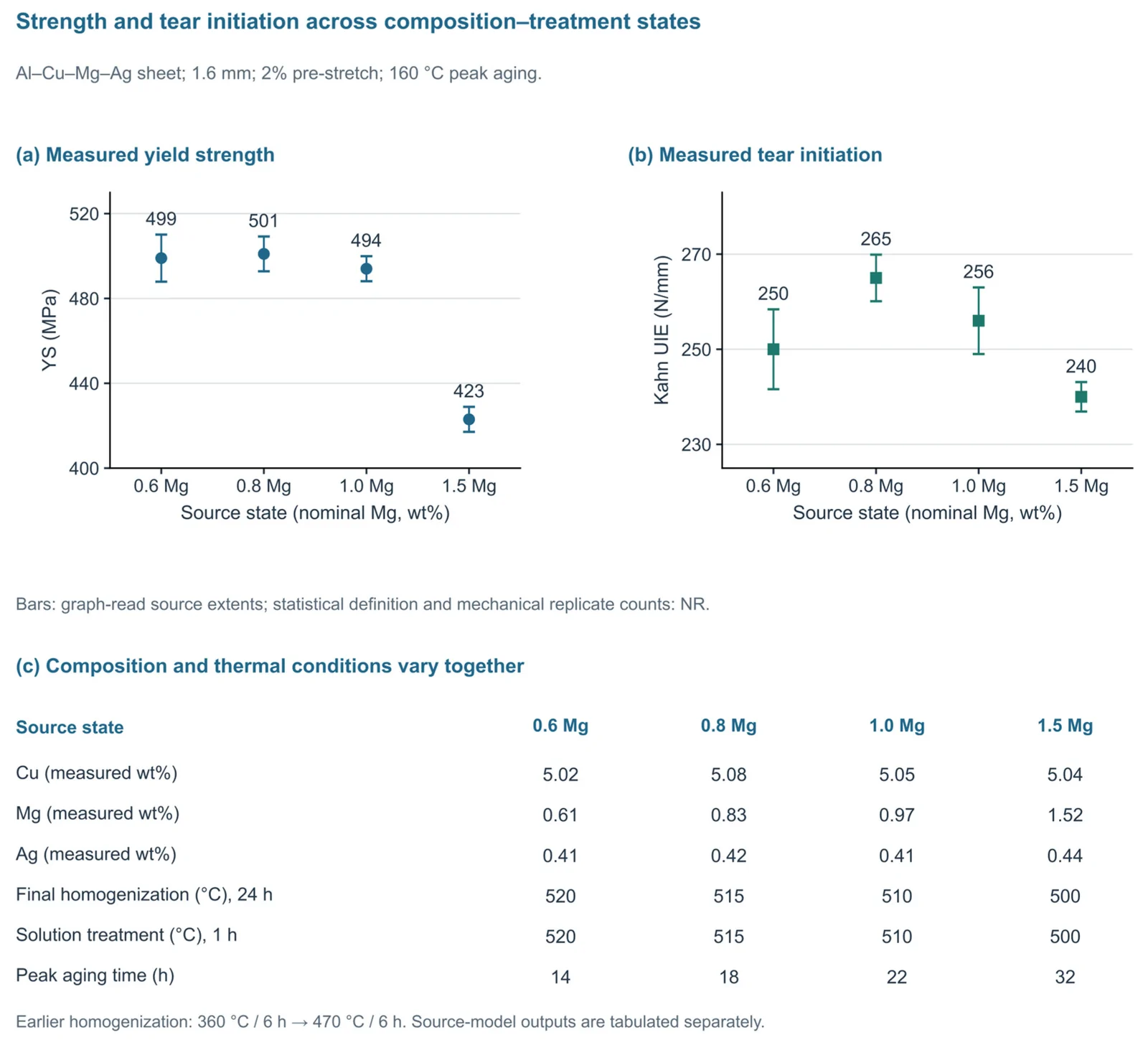
Measured strength and tear initiation across four composition–treatment states [31]. (**a**) Yield strength (YS); (**b**) Kahn unit initiation energy (UIE), defined as energy to maximum tear load divided by initial ligament area; (**c**) measured Cu/Mg/Ag contents and thermal schedules. The category labels denote nominal Mg contents, not a continuous single-variable experiment. All sheets were 1.6 mm thick, water quenched, pre-stretched by 2%, and peak-aged at 160 °C; the homogenization, solution temperature, and peak-aging time covary. The reproduced graphical error bars have unreported statistical definitions and specimen counts, and are not assigned standard deviation (SD), standard error (SE), or confidence interval (CI) meanings. Extraction details are in Methods S1. UIE is not K_IC_; source-model quantities are separated in Table S2. Possible baseline overlap with [44] is retained in the source records.

The four composition–treatment states in Figure 1 show a non-monotonic strength–tear balance: the highest nominal Mg state has a lower yield strength and unit initiation energy, while the three lower-Mg states are closer in strength [31]. Homogenization and solution temperatures and peak-aging duration also change across the series. The appropriate comparison is therefore among composition–thermal-schedule combinations.The small separation between the leading strength values does not establish a statistical optimum.

The source-model K* and Ω strengthening share are reported separately in Table S2 [31]. K* is a model-derived toughness indicator and not measured K_IC_, while the Ω share is a contribution to modeled precipitation strengthening and not phase volume fraction. The measured YS/UIE bars in Figure 1 graphically reproduce the source half-spans; their statistical meaning and mechanical specimen counts remain NR. Together with Mg-dependent corrosion evidence [32], the four composition–treatment combinations support screening complete states by several endpoints rather than ranking chemistry by strength alone.

The separate roles of Cu, Mg, and Ag become clearer when the comparison unit is specified. In a cast Al–4.6Cu–0.3Mg series, Ag additions changed the balance of Ω and θ′ precipitation and hardness under the investigated T7 schedules [33]. Quantitative TEM/APT work on another series linked increasing Ag to denser Ω precipitation, greater enrichment in the precursors, and the suppression of θ′ [34]. A later APT study resolved stronger Mg–Ag co-clustering with increased Ag, and treated the resulting order-hardening contribution as a calculation [35]. These findings support Ag-assisted precipitation under the stated solute balance; they neither isolate the behavior of a processed commercial sheet nor demonstrate a fatigue benefit.

Mg changes both precursor kinetics and the competition for solutes. The early-aging comparison at 165 °C found a non-monotonic population of Mg–Ag co-clusters across 0.39, 0.81, and 1.18 wt% Mg [36]. The low residual cluster count in the highest-Mg state was interpreted partly as faster progression toward Ω, illustrating why a snapshot cluster population is not a direct measure of the eventual precipitate population. In a different wrought Al–4Cu–xMg–0.6Ag series, greater hardness coexisted with a reduced yield response and ductility, while the electrochemical behavior changed with the S-phase population [37]. These are different metrics and states and not a common Mg optimum.

An Ag/no-Ag comparison provides a useful within-study contrast. Tai et al. examined Al–5.1Cu–1.0Mg with 0 or 0.4 wt% Ag at 185 °C; both states peaked after 11 h, and the Ag-bearing state showed a higher tensile strength and slower subsequent strength loss alongside Ω/S competition [38]. This strengthens the phase-competition interpretation but does not make the pair an AA2139 fatigue experiment. Increasing Cu likewise changes more than the available amount of Ω-forming solute; a 2.77–6.80 wt% Cu series showed changes in constituent particles, recrystallization, boundary precipitation and a non-monotonic Ω population [39]. Its displayed number-density unit is unresolved in the source record, so no cross-paper numerical density ranking is made here. A narrower Cu series additionally measured crack propagation and corrosion, as discussed in Section 5.4 [40].

Early clustering and interfacial descriptions are complementary rather than interchangeable. Atom probe observations during the first seconds of 180 °C aging [41] should not be compared as if they described an as-quenched specimen; that paper reported atomic-percent compositions, unlike weight-percent engineering comparisons. Positron spectroscopy provides separate evidence concerning vacancy trapping and release during multi-step aging [42]. The structural reinterpretation of Ω using density functional theory (DFT) [43] concerns lattice symmetry and matrix constraint, whereas measured phase dimensions, fractions, and mechanical tests answer different questions. Together, these studies justify tracking solute, defect, and interface states separately, with Table 2 organizing the practical comparison limits.

**Table 2:** Composition–history comparison units. Concentrations are in wt% unless explicitly identified otherwise; fuller source-specific conditions are retained in Table S1.

Comparison	Chemistry	State/Processing	Supported Endpoint/Limit
Four Mg–treatment states [31]	Measured Cu ≈ 5 wt%; Mg and Ag listed in Figure 1	1.6 mm sheet; homogenization, solution temperature and peak time covary	YS and Kahn UIE; no pure Mg causal effect
Ag-controlled comparisons [33,34,35]	Cast Al–4.6Cu–0.3Mg and separate APT alloy series	T7 or early/peak aging under each source schedule	Hardness/precipitation; no common fatigue endpoint
Ag/no-Ag pair [38]	Al–5.1Cu–1.0Mg with 0/0.4 wt% Ag	185 °C; 11 h peak, 18/100 h for later states	Ω/S competition and tensile retention; not AA2139 fracture
Cu-controlled series [39,40]	2.77–6.80 wt% Cu; separate 3.19/3.51/3.78 wt% series	Different processing and peak-aging states	Tensile/phase data; second series adds fatigue crack growth (FCG)/intergranular corrosion (IGC); no pooled response
Homogenization pair [44]	Al–5.02Cu–0.61Mg–0.41Ag, with Mn/Zr	2 mm sheet; single-stage/triple-stage homogenization (SH/TH), followed by common downstream processing	Tensile and phase descriptors; possible baseline overlap with [31]
Industrial-ingot homogenization [45]	Bottom D/4: Al–5.23Cu–0.94Mg–0.62Ag, with 0.66Mn/0.12Zr (wt%)	420 °C/5 h + 480 °C/8 h; then 510 or 515 °C/24 h; WQ	Scanning electron microscopy with backscattered-electron contrast (SEM–BSE); residual constituents; not peak-aged Ω or a mechanical/damage endpoint
Cross-over boundary [46,47,48,49,50]	Other alloy system: Al–Mg–Zn–(Cu,Ag); phase identity source specific	Low-temperature storage, single/two-step aging, or high-temperature forming	T/cluster or η′ evidence; no assumed Ω-alloy damage-tolerance equivalence

### 3.3. Homogenization and Rolling Leave a Persistent Structural Inheritance

**Figure 2:**
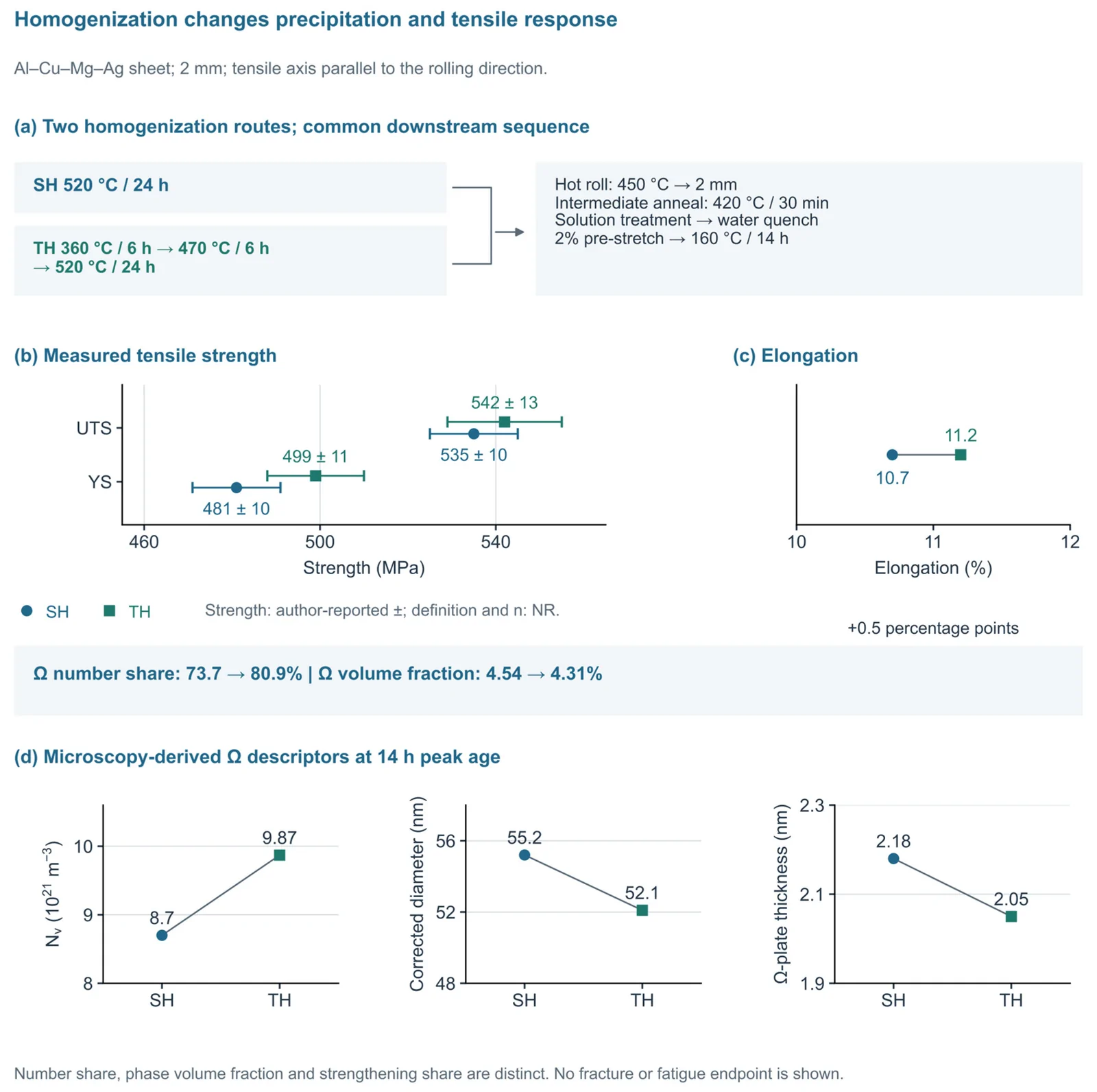
Homogenization-dependent precipitation and tensile response in 2 mm Al–Cu–Mg–Ag–Zr sheet [44]. (**a**) Single-stage (SH) and triple-stage (TH) homogenization, followed by common downstream processing, 2% pre-stretch, and 160 °C/14 h aging. Hot rolling is shown at 450 °C following the source text; the conflicting schematic is documented in Methods S1. Solution-treatment temperature and duration are NR. (**b**) YS and UTS, with source-reported ± values of unspecified statistical meaning; tensile n is NR. (**c**) Elongation, with conflicting SH/TH error assignments omitted. (**d**) Ω number density, corrected diameter and plate thickness in the peak-aged state; number share (Ω/total counted Ω, θ′, and S precipitates) and volume fraction remain distinct. These are tensile and microscopy endpoints. Identical TH and Ref. [31] tensile central values do not establish independent replication because their specimen linkage remains unresolved.

Homogenization changes the distribution of solute and constituent particles before the forming sequence begins. In the comparison summarized in Figure 2, single-stage and triple-stage homogenization were followed by common rolling, solution treatment, water quenching, 2% prestrain, and aging at 160 °C for 14 h [44]. Yield strength increased from 481 ± 10 to 499 ± 11 MPa and ultimate tensile strength from 535 ± 10 to 542 ± 13 MPa; the reported ± definitions were not specified. Elongation increased from 10.7% to 11.2%, a difference of 0.5 percentage points. These tensile changes characterize the final state produced by the complete sequence; fracture improvement was not measured.

The associated Ω descriptors reveal why different metrics should not be collapsed into a single precipitate-content variable. Number density increased from 8.7 to 9.87 × 10^21^ m^−3^, while the consistently corrected diameter decreased from 55.2 to 52.1 nm and thickness from 2.18 to 2.05 nm [44]. The Ω number share, defined as the number of Ω precipitates divided by the total number of Ω, θ′, and S precipitates counted in this comparison, increased, but its reported volume fraction decreased slightly. More numerous, smaller plates can therefore coexist with a lower phase volume fraction. Because the 0.6 wt% Mg state in Figure 1 and the triple-homogenized state in Figure 2 share identical tensile central values despite different stated sheet thicknesses, they are not treated here as independent replication; the specimen lineage requires clarification.

Rolling and recrystallization also influence the inherited state through the grain shape, texture, dispersoids, and strain partitioning. Reviews of anisotropic plasticity and texture-based forming simulations show why one uniaxial tensile direction cannot calibrate a complete sheet-forming response [51,52]. A recent Al–Cu–Mn–Mg–Ag processing study used bimodal grains and multi-scale particles to improve the tensile strength–ductility combination [53]. Such architectures are relevant candidates for controlling localization, but the tensile result alone cannot establish resistance to long-crack propagation. The forming study must retain the rolling direction, through-thickness position, and particle distribution as part of the initial condition that will later be linked to crack paths.

**Figure 3:**
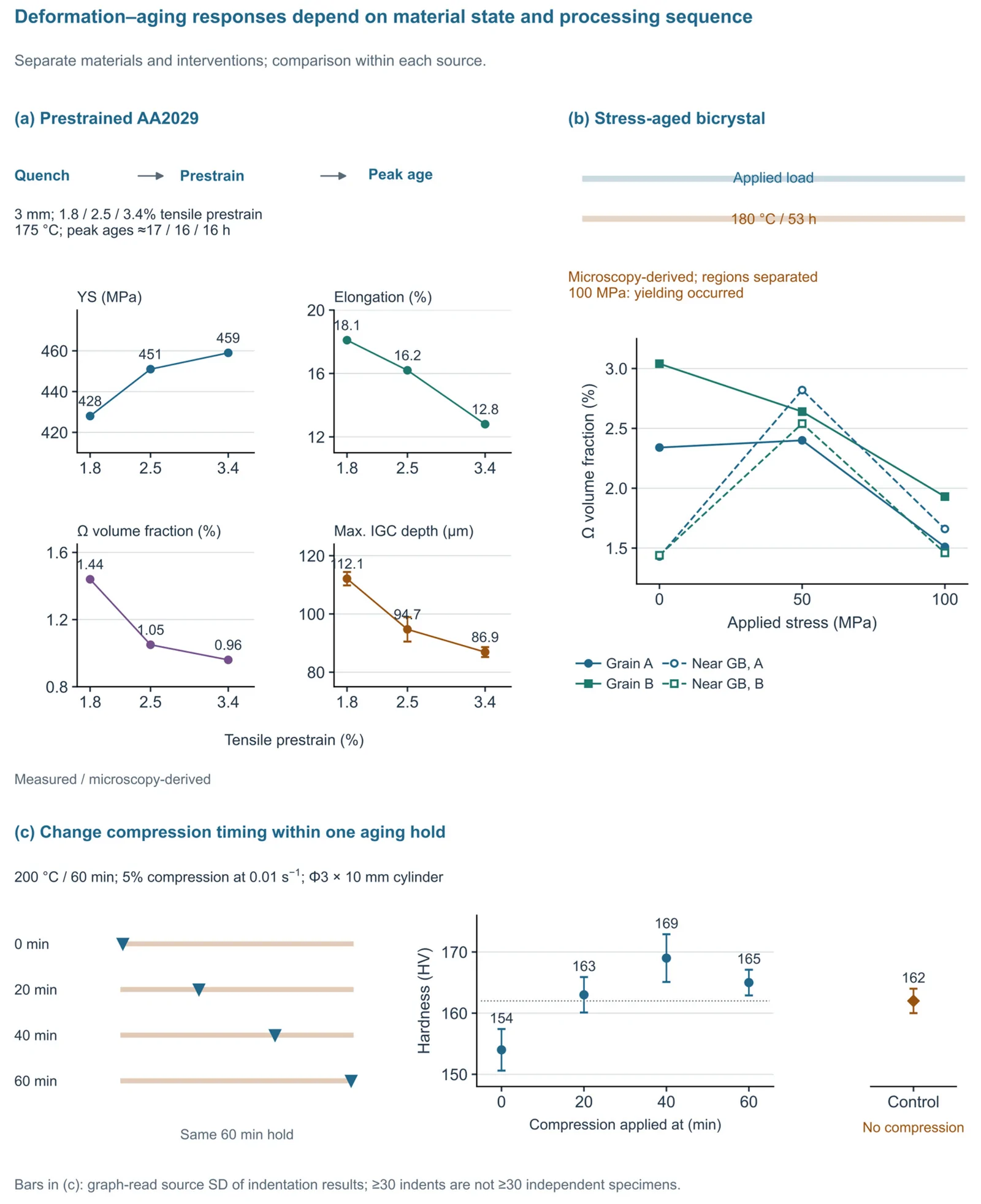
Material states and sequence dependence of deformation–aging responses in three separate experiments. (**a**) YS, elongation, Ω volume fraction, and maximum intergranular-corrosion depth for 3 mm AA2029 sheet after 1.8/2.5/3.4% tensile prestrain and 175 °C peak aging [58]. Prior treatment was 513 °C/1.5 h and water quenching; approximate peak times were 17/16/16 h. The source reports 3–5 parallel tensile tests without state-specific n. IGC followed 6 h exposure at 35 °C; its ± definition and independent n are unspecified. (**b**) Grain-interior and near-boundary Ω volume fractions in Al–1.96Cu–0.24Mg–0.18Ag bicrystals after 525 °C/2 h, water quenching and 180 °C/53 h aging under 0/50/100 MPa [68]; yielding occurred at 100 MPa. Lines guide the eye. (**c**) Five-percent compression at 0/20/40/60 min of a 200 °C/60 min hold versus an uncompressed control [73]. Cylinders (Φ3 × 10 mm) were machined from 3 mm sheet, treated at 515 °C/2 h and naturally aged for four days; compression rate was 0.01 s^−1^. Bars reproduce source indentation SD; at least 30 indents do not establish 30 independent specimens. Equal-length strips indicate timing and not compression duration. The separate APT and STEM/EDS–DFT sources [61,62,74] retain their own conditions and evidence roles. Source conflicts and graphical extraction details are in Methods S1.

**Figure 4:**
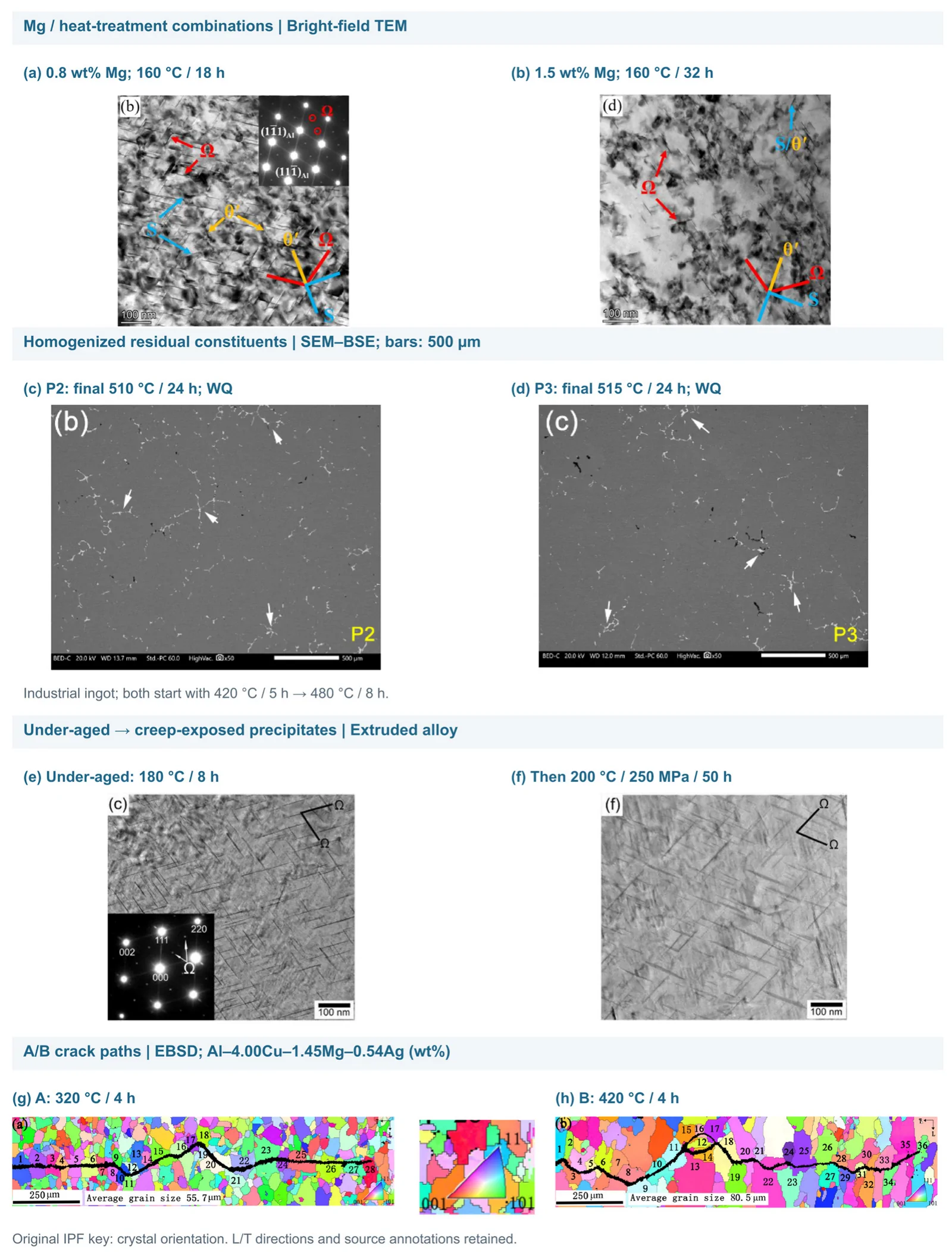
Source-specific microscopy of composition–treatment states, homogenized residual constituents, creep-exposed precipitates, and fatigue crack paths. (**a**,**b**) Bright-field TEM of <110>Al for the nominal 0.8/1.5 wt% Mg states [31] (original Figure 9b,d); measured Cu/Mg/Ag contents are 5.08/0.83/0.42 and 5.04/1.52/0.44 wt%. The 1.6 mm sheets received 2% pre-stretch and 160 °C peak aging for 18/32 h; solution treatments were 515/500 °C for 1 h, with composition-specific homogenization. (**c**,**d**) Scanning electron microscopy with backscattered-electron contrast (SEM–BSE) images from the bottom D/4 position of an industrial Al–Cu–Mg–Ag ingot [45] (original Figure 5b,c). P2 and P3 share 420 °C/5 h and 480 °C/8 h stages, followed by 510 or 515 °C/24 h, respectively, and water quenching. These are residual second-phase observations after homogenization, not peak-aged Ω precipitates. (**e**,**f**) Near-<110>Al bright-field TEM of the 6.00 wt% Cu, Sc/Zr-containing extruded alloy [54] (original Figure 2c,f) after under-aging at 180 °C/8 h and after subsequent creep at 200 °C/250 MPa/50 h. The pair combines additional thermal exposure and loading; it is not an unloaded/loaded comparison at identical thermal history. No same-specimen or same-field pairing is inferred. (**g**,**h**) EBSD crack paths of 5 mm Al–4.00Cu–1.45Mg–0.54Ag–0.52Mn A/B specimens [16] (original Figure 7a,b), corresponding to Figure 6b. The 320/420 °C, 4 h pre-anneals were followed by 490 °C/20 min, water quenching and natural aging. The unchanged inverse pole figure (IPF) key encodes orientation; original L/T directions and grain labels are retained. Scale bars: 100 nm (**a**,**b**,**e**,**f**), 500 µm (**c**,**d**), and 250 µm (**g**,**h**). Phase arrows and diffraction labels are original. Only rectangular cropping and proportional placement were applied; comparisons remain within each source. Additional processing conditions are in Table S1. Image credit: (**a**,**b**) Wang et al. [31], DOI:10.1016/j.jmrt.2026.03.038; these panels are retained following editorial confirmation. (**c**,**d**) Lin et al. [45], *Materials* 2023, 16, 433, DOI:10.3390/ma16010433, © 2023 the authors, CC BY 4.0 (https://creativecommons.org/licenses/by/4.0/, accessed on 29 September 2026). (**e**,**f**) Peng et al. [54], *Metals* 2021, 11, 487, DOI:10.3390/met11030487, © 2021 the authors, CC BY 4.0 (https://creativecommons.org/licenses/by/4.0/, accessed on 29 September 2026). (**g**,**h**) Hu et al. [16], *Materials* 2018, 11, 2481, DOI:10.3390/ma11122481, © 2018 the authors, CC BY 4.0. Cropping and proportional placement only; review panel labels and condition headings were added outside the source images.

Figure 3 presents the subsequent deformation–aging comparisons, while Figure 4 provides source-specific microscopy across distinct processing states. Panels (a,b) show two composition–treatment combinations from [31]. Panels (c,d) compare the residual second phases after two homogenization schedules in an industrial Al–Cu–Mg–Ag ingot [45]; they do not reproduce the peak-aged SH/TH comparison quantified in Figure 2. Panels (e,f) show the under-aged state and the state after creep exposure in an extruded Sc/Zr-containing Al–Cu–Mg–Ag alloy [54]. This pair includes both additional thermal exposure and loading rather than isolating the applied-stress effect quantified for bicrystals in Figure 3b. Panels (g,h) retain the A/B electron backscatter diffraction (EBSD) crack-path maps from the FCG study [16]. The unchanged inverse pole figure (IPF) color key encodes crystal orientation, not phase or stress. Full source-panel mappings and scale bars are given in the caption. Table 2 and Table S1 preserve the relevant chemistry, forms, and initial states.

### 3.4. Cross-Over Alloys as a Bounded Comparison of Forming–Hardening Strategies

Al–Mg–Zn–(Cu,Ag) cross-over alloys are used here as comparisons from a different alloy system. Ceci et al. review Al–Mg–Zn–(Cu) alloys, including T-phase strengthening and emerging applications [55]; a broader snapshot review relates this approach to the strength–formability and corrosion balances of Al–Mg-based products [56]. Here, the comparison is restricted to composition, clustering, forming state, and subsequent hardening. Radiation performance does not answer the present sheet-forming and crack-resistance questions.

Stemper et al. added Zn to an Al–Mg base and examined Cu and Ag additions by hardness, tensile testing, and microscopy [46]. T-phase precipitation and its precursors provided age hardening while the soft state retained useful strain-hardening capacity. Ag enhanced the response, but its effect depended on whether single- or two-step aging was used. The relevant analogy to Ω-strengthened sheet is the deliberate separation of a formable starting state from the strengthened state; the phase identity, solute balance, and thermal schedule remain different.

Recent primary results also prevent a universal favorable Cu/Ag conclusion. In the over-aging study of Willenshofer et al., single-step aging at 125 °C and a two-step route ending at 175 °C produced different strength–corrosion combinations; Cu/Ag increased the strength but reduced corrosion resistance within the examined conditions [47]. The more general corrosion advantages discussed in the cross-over review [56] therefore require their own composition and temper qualifiers. They cannot be transferred to all over-aged alloys. A separate low-temperature study used 60 °C aging over 42 days to examine clustering and mechanical response [48]. It supports the importance of storage and cluster evolution, but its duration is not a practical substitute for a short forming or paint-bake cycle.

Processing can also change the role of a particle population during the route. Samberger et al. used coarse T-phase particles to stimulate recrystallization in Al–Mg–Zn–Cu sheet [49]. The source’s Table 2 separates recrystallization at 380 °C/5 min from solution annealing at 480 °C/1 min. One stability route applied 470 °C for approximately 24 h after solution annealing and water quenching; a separate age-hardenability sequence used 480 °C/1 min, water or compressed-air quenching, 100 °C/5 h pre-aging, 14 days at room temperature and 185 °C/20 min paint baking. The study demonstrates grain refinement, high-temperature stability and subsequent hardening through distinct comparisons. These routes are not combined here into a single forming-then-solution-treatment chain or treated as a 160–200 °C creep-age-forming (CAF) analog. In a different automotive Al–Mg–Zn–Cu sheet study, pre-aging and paint-bake responses were discussed through Guinier–Preston (GP) zone and η′ evolution and forming-limit measurements [50]; the chemical family name alone does not justify relabeling this microstructure as T-phase dominated. None of these comparisons establish matched long-crack growth or residual strength for an Ω-strengthened formed skin. Their contribution is to show which starting-state and sequence controls can be shared across alloy design strategies, as well as which phase-specific claims require separate verification.

## 4. Deformation–Aging Order and the Control of Local States

### 4.1. Plastic Prestrain Before Aging

Prestrain introduces dislocations before the principal artificial-aging treatment. Its effect is therefore not restricted to direct work hardening, as it can change heterogeneous nucleation, solute competition, and the spatial distribution of subsequent precipitation. Gazizov and Kaibyshev found that deformation before aging modified competition between Ω and dislocation-associated precipitates in an Al–Cu–Mg–Ag–Mn–Sc–Zr alloy [57]. The largest rolling reductions also introduced pronounced structural and mechanical anisotropy. This provides mechanistic support for a sequence effect, but severe rolling reductions must not be treated as equivalent to the few-percent strains typically used as laboratory proxies for sheet forming. Figure 3 distinguishes prestrain before aging from loading during aging.

The AA2029 experiment in Figure 3a provides a more directly relevant small-prestrain comparison [58]. The 3 mm sheet contained Al–3.62Cu–0.90Mg–0.45Ag (wt%) and was water quenched after solution treatment at 513 °C for 1.5 h. Immediate tensile prestrains of 1.8%, 2.5%, and 3.4% were followed by peak aging at 175 °C. Figure 2 of Ref. [58] presents the corresponding approximate strength-peak times of 17, 16, and 16 h; the exact aging duration assigned to each characterized coupon is not tabulated. Yield strength increased from 428 to 451 and then to 459 MPa, while elongation decreased from 18.1% to 16.2% and then to 12.8%. The increasing strength is therefore accompanied by a substantial loss in tensile ductility. Neither trend, separately or together, establishes the ranking of fracture toughness or fatigue-crack-growth resistance.

The microstructural response helps explain this decoupling. Across the same prestrain series, the reported Ω volume fraction decreased from 1.44% to 1.05% and then to 0.96%, although the yield strength increased [58]. The Ω number densities were 4.09, 2.60, and 3.01 × 10^21^ m^−3^ and were therefore not monotonic. S-phase precipitation and deformation-induced dislocations provide plausible additional strengthening contributions; the measured trends do not isolate their relative magnitudes. The authors interpreted the changes partly through vacancy consumption and competition involving Mg–Ag clusters. Because this cluster explanation draws on earlier research rather than direct cluster measurements in this experiment, it is retained here as an interpretation of the observed precipitate changes.

The accompanying immersion test found lower maximum intergranular-corrosion depths with increasing prestrain, from 112.1 ± 2.3 to 94.7 ± 4.2 and then to 86.9 ± 1.7 μm after 6 h at 35 °C in NaCl/H_2_O_2_ solution [58]. This environment-sensitive response is associated with the processed grain-boundary state. It is not a stress-corrosion-cracking or corrosion-fatigue result. Furthermore, the paper contains conflicting statements about precipitate-free-zone (PFZ) width, so a monotonic narrowing of this zone is not used to close the mechanistic argument. The defensible result is a coupled change in measured tensile properties, precipitation and immersion-corrosion penetration, with the fracture and fatigue consequences still to be determined.

The newer pre-deformation studies sharpen this distinction. In AA2139 plates, equal nominal percentages of cold stretching and cold rolling produced different equivalent-strain fields and surface responses [59]. Rolling introduced a through-thickness gradient, so a nominal 7% rolling reduction is not mechanically equivalent to 7% tensile extension. A subsequent 5–15% rolling series reported finer, more numerous Ω/θ′ precipitates and faster peak aging, with ductility deteriorating at the largest reduction [60]. Ref. [59] defines its tensile “toughness” as the engineering stress–strain curve area and reports relative improvements. Ref. [60] explicitly integrates engineering stress over engineering strain and reports tensile work per initial volume in MJ/m^3^. These tensile curve metrics are distinct from fracture energy per crack area or fracture toughness; Table S1 preserves their source definitions. These two reports have a related research and product lineage and are not counted as independent replication of a universal prestrain rule.

Their strength improvements need not invalidate the decreased initial age hardening in small-stretch APT studies [61,62]. The latter directly observed suppressed Mg–Ag co-clustering under their own tensile-prestrain and aging conditions. The composition, strain mode, magnitude, storage history, and sampled aging stage all differ. Earlier comparative cold-work experiments already showed that morphologically similar Ω and T1 plates can respond differently to deformation in Ag-bearing and Li-bearing alloys [63]. Thus, a shared plate habit does not establish a shared nucleation response. The available studies leave the relative roles of these changed conditions unresolved; a controlled common-alloy test is needed to apportion them.

Early versus late loading provides a more specific mechanistic contrast. Two-step stress/free-aging experiments linked the stress-oriented Ω response primarily to the early nucleation stage [64]. An Ag-free Al–Cu–Mg bicrystal exhibited boundary-side-dependent S-variant populations and hardness under stress aging [65]. This adjacent-system result supports spatial sampling around boundaries, but it cannot supply an Ω volume fraction or a critical stress for AA2139. Together with Figure 3, these studies support recording the time of loading, local orientation and onset of plasticity before assigning an apparent stress effect to a particular precipitate mechanism.

The paired microscopy observations in Figure 4 place composition–treatment combinations, homogenized residual constituents, and creep-exposed precipitate structures in their respective material and observation conditions. Its EBSD crack-path pair is considered with the separate long-crack experiments in Section 5.2.

### 4.2. Loading During Aging: Stress, Plasticity and Grain-Boundary Proximity

Loading during aging acts on evolving precipitation and defect structures. Single-crystal studies demonstrate orientation-dependent Ω populations and competing precipitation under applied stress [66,67]. To compare such results, stress sensitivity must be resolved into variant selection, precipitate volume or number density, dimensions, and kinetic changes. The same nominal stress may affect one observable without producing an equivalent change in another, particularly when the initial mechanical state and grain orientation differ.

The dilute bicrystal experiment in Figure 3b compares grain interiors with regions adjacent to the grain boundary [68]. Al–1.96Cu–0.24Mg–0.18Ag material was solution treated at 525 °C for 2 h, water quenched, and aged at 180 °C for 53 h under 0, 50, or 100 MPa. At 50 MPa, the Ω volume fractions near the two sides of the boundary increased from 1.43% and 1.44% to 2.82% and 2.54%, respectively. The grain interiors did not follow one common response: the corresponding values for grains A and B changed from 2.34% and 3.04% to 2.40% and 2.64%. Averaging these regions would obscure the spatial effect that makes the experiment informative.

At 100 MPa, yielding occurred in the initial solution-treated state, and the Ω volume fractions were lower than at 50 MPa in all four reported regions [68]. The higher load is consequently not a purely elastic stress-aging condition. TEM estimates also showed increased dislocation density in grain B and its near-boundary region. For forming, this distinction matters because nominal load can cross a changing yield surface during the thermal cycle, so an experiment labeled stress aging may contain different amounts of plastic deformation. Bicrystal compression experiments and crystal-plasticity simulations independently illustrate the role of orientation mismatch and slip transfer in local deformation [69]. They motivate a spatially resolved analysis without providing a universal conversion from applied stress to retained damage tolerance.

Bakavos et al. provide a low-stress comparison [70]. In T351 material subsequently aged to T8, weak residual stresses remaining after stretching biased Cu GP-zone and θ″ habit-plane selection in AA2022, whereas the dominant Ω population in AA2139 showed no corresponding variant bias. Their supplied products included 40 mm plate and 5 mm sheet, and the residual-stress interpretation concerned thick plate. This result does not contradict the bicrystal change in Ω volume fraction: the interventions and observables differ. Absence of variant selection under weak residual stress is not evidence of an unchanged volume fraction under a higher external load. Conversely, a change in total Ω volume fraction does not establish variant selection. The variables needed for comparison are stress relative to the evolving yield level, initial defects, orientation and measurement location. An engineering-alloy stress-aging study independently reports reduced initial hardening and a shift toward θ′ precipitation at the expense of Ω under applied stress or prior deformation [71]. The full text identifies a 3 mm polycrystalline alloy aged at 170 °C and a separate 6% pre-stretch comparison. These observations support precipitation competition under those histories; they do not define a transferable stress or strain threshold.

**Figure 5:**
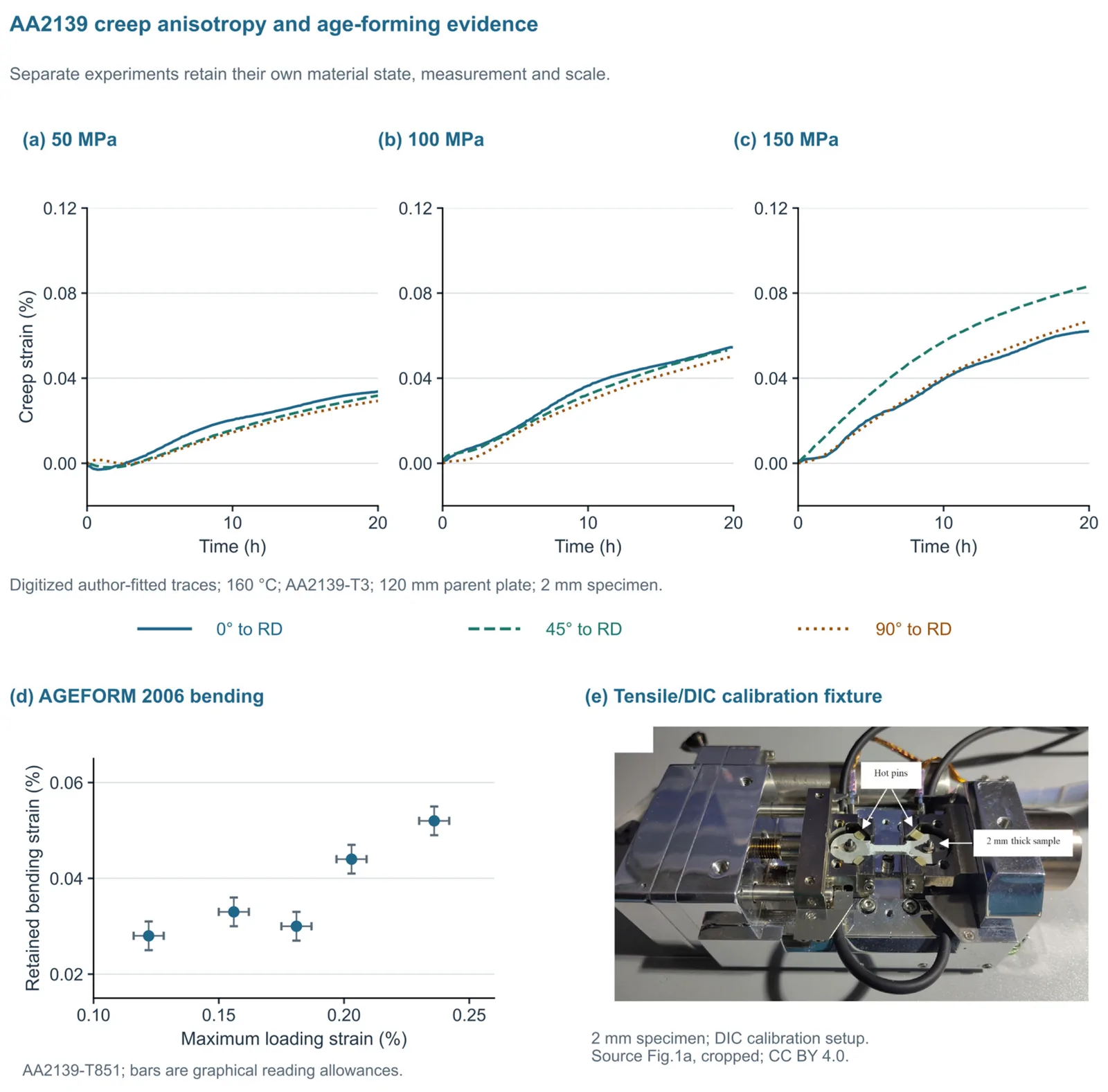
Source-specific creep and age-forming evidence for AA2139. (**a**–**c**) Author-fitted DIC creep traces at 160 °C under initial stresses of 50/100/150 MPa along 0°/45°/90° to RD [72] (source Figure 5). AA2139-T3 specimens were 2 mm thick and extracted near the surface of a 120 mm parent plate. The 1809 digitized coordinates describe nine fitted traces and not independent tests. (**d**) Five AA2139-T851 markers from Figure 43 of the 2006 AGEFORM report [8], comparing maximum loading strain with retained bending strain. Bars are graphical reading bounds (±0.006/±0.003 percentage points horizontally/vertically) and not experimental uncertainty. T851 is retained as the source label. (**e**) Tensile-stage specimen clamping for DIC calibration [72] (source Figure 1a) and not a CAF bending device. Image credit: Gautier et al. [72], DOI: 10.4028/p-x19oza, CC BY 4.0; cropped and proportionally resized. Independent n per configuration is NR. The creep and bending data have separate specimen and processing histories; extraction details are in Methods S1.

Engineering AA2139 adds a complementary observation. Gautier et al. extracted specimens near the surface of a 120 mm T3 plate and combined creep aging at 160 °C with digital image correlation (DIC) and in situ electron backscatter diffraction (EBSD) [72]. Tests at 50, 100, and 150 MPa and at 0°, 45°, and 90° to the rolling direction showed that directional creep rankings changed with load (Figure 5). Normalized mean kernel average misorientation (KAM) approached saturation after about 10 h. KAM quantifies the mean local misorientation between an EBSD measurement point and selected neighboring points; it is neither a distance-normalized orientation gradient nor a phase-fraction measurement. Interpretation depends on acquisition and analysis settings. Here, the scan step was 1 µm and each test’s mean KAM was normalized by its own maximum. Assigning the trend to a particular precipitate population remains an interpretation without phase-resolved TEM or diffraction in this experiment. Local sampling should therefore retain both direction and stress history. This thick-plate-derived experiment supplies an engineering bridge from controlled crystals to polycrystals, with repeatability still identified by its authors as a priority.

The timing experiment of Tai et al. [73] provides an additional within-source comparison rather than another tensile-prestrain series. Cylindrical compression specimens (Φ3 × 10 mm) were machined from 3 mm Al-5.13Cu-0.97Mg-0.38Ag sheet and received 515 °C/2 h treatment with four days of natural aging. Five-percent compression at 0.01 s^−1^ was applied at 0, 20, 40, or 60 min within a 200 °C/60 min hold. The respective source-text hardness means are 154, 163, 169, and 165 HV, compared with 162 HV for the separate uncompressed control (Figure 3c). The graphically restored bars are source-indentation SDs; at least 30 indents on one specimen do not establish 30 independent specimens. The sequence produces a non-monotonic hardness response within these conditions. It does not establish a tensile-prestrain optimum, CAF schedule, or fatigue advantage.

The AA2029 cluster mechanism [58] and the direct APT observations in separate small-prestrain studies [61,62] concern different experiments. Qin et al. [74] add a processing-order comparison using microscopy and tensile tests; their scanning transmission electron microscopy (STEM) and energy-dispersive X-ray spectroscopy (EDS) observations and DFT calculations have distinct roles. Section 4.3 considers what this comparison adds to the sequence argument.

### 4.3. Pre-Aging, Heating Rate and the Persistence of a Starting State

Pre-aging changes the state that receives deformation and can therefore change the meaning of an otherwise identical prestrain. In an Al–6.5Cu–0.4Mg–1.6Ag sheet study, pre-aging before 2.5% stretching modified precipitation and the final tensile response [75]. The complete schedules are retained in Supplementary Figure S2: different pre-aging temperatures were followed by different durations of final aging at 165 °C. The comparison establishes schedule-specific properties and not the isolated effect of pre-aging temperature. Mechanistically, pre-existing clusters or precipitates alter the competition faced by deformation-generated defects; operationally, the material must be described by the actual thermal and storage history at the instant deformation begins.

Qin et al. compared direct 5% tensile pre-deformation followed by aging with a route that inserted 100 °C/6 h pre-aging before the same nominal stretch in 2 mm Al–Cu–Mg–Ag sheet [74]. Their TEM/STEM observations showed a denser Ω distribution in the pre-aged route, while the reported tensile comparison favored its strength response over direct pre-deformation; subsequent exposure at 200 °C also produced a smaller loss of UTS. In source Figure 10c–f,h, microscopy contrast and EDS maps/line scans, after pre-aging and before stretching, support nano-scale Mg/Ag-enriched regions that the authors identified as Mg–Ag co-clusters. This microscopy and local chemical evidence is distinct from the APT-based cluster analyses in [61,62]. DFT supports the authors’ proposed vacancy-related stabilization mechanism; it does not measure cluster survival through the subsequent stretch. The source’s 4 h/6 h peak-aging assignments remain unresolved, so the tensile and final Ω observations compare complete reported routes and do not isolate a causal effect of the 100 °C/6 h step alone. The comparison supports considering the state before deformation, but it does not establish K_IC_, fatigue crack-growth resistance, or a fatigue benefit.

The heating rate is another state variable. Non-isothermal aging experiments in Al–Cu–Mg–Ag showed that ramp conditions changed Ω size distributions, precipitation kinetics and the peak-aged tensile response [76]. A nominal aging temperature therefore omits potentially important exposure accumulated during heating and transfer. The same concern applies to creep. Long-duration creep studies found distinct sensitivities of Ω and dislocation-associated S/θ′ complexes to exposure time and deformation [77]. Peng et al. [54] provide a separate before/after example: the 6.00 wt% Cu alloy, containing Sc and Zr and processed as extruded bar, was under-aged at 180 °C for 8 h and then crept at 200 °C and 250 MPa for 50 h. Figure 4e,f retains the corresponding precipitate observations. Their comparison reflects the combined thermal and mechanical exposure and not a stress-only effect or direct validation of sheet creep-age forming. Such results argue for recording the full local time–temperature–stress path. They do not justify extrapolating a long-term creep response to a short forming cycle without validating the relevant kinetic regime.

Ω plates can interact with dislocations through more than one mechanism. Atomic-resolution observations after creep at 150 °C identified shearing of thinner plates and dislocation loops around thicker plates, with both interactions observed in the transition range [78]. Subsequent work examined the slip systems of both Ω′ and Ω using microscopy, image simulation, and first-principles calculations [79]. These observations weaken any simple assumption that maximizing Ω content automatically suppresses all deformation localization. The useful descriptors include plate thickness and spacing, phase identity, and interfacial state, together with the matrix slip geometry. Linking those descriptors to crack-tip cyclic plasticity remains a separate experimental task.

Experiments involving severe deformation further delimit the range of transfer. Room-temperature equal-channel angular pressing changed precipitation competition in AA2139 and produced behavior different from an Ag-free comparison alloy [80]. Large predeformation in an Al–Cu–Li sheet likewise increased creep formability while intensifying directional differences [81]. These studies are valuable for identifying possible mechanisms and for challenging monotonic assumptions. Their deformation magnitudes, grain structures and, in the latter case, strengthening system are substantially different from the target small-strain sheet route. They are used as boundary cases, not as numerical prescriptions for fuselage-skin forming.

Additional schedules show why peak hardness is only one selection criterion. Pre-aging, 40% cold rolling, and re-aging improved the tensile balance in an AA2139 study [82]; the large reduction and complete thermal route distinguish it from small-strain skin forming. Under-aging improved creep resistance at 130–150 °C in another Al–Cu–Mg–Ag experiment, but subsequent ambient storage progressively removed this benefit [83]. An over-aging study instead traded some room-temperature strength for improved retention during later heat exposure [84]. These results concern tensile response, creep and thermal retention. They motivate recording the waiting interval and intended exposure, rather than selecting a single peak-aged state for every endpoint. Recent slip-transmission analysis combining high-angle annular dark-field STEM (HAADF-STEM), crystallography, and atomistic calculations further linked the mechanical interaction with Ω to plate geometry [85]; a calculated transmission preference is not a measured crack-growth law.

### 4.4. Forming Routes as Different Sequences of State Change

Forming routes impose different sequences of state change. Table 3 compares prestraining before aging, loading during aging, and creep-age forming in terms of their initial states, processing conditions, microstructural observations, measured responses, and limits of interpretation. Post-quench forming makes the quench-to-form delay, local plastic strain, and subsequent aging schedule central controls. The AA2029 and related sheet experiments establish the relevant state changes [31,44,58]. CAF additionally couples time-dependent strain and stress redistribution to ongoing precipitation. Figure 5 emphasizes direct AA2139 forming and loaded-aging evidence, while Supplementary Figure S3 organizes the alternative route sequences and their method references. A type-2024 patent describes F/W/T3/T4 forming and heat-treatment variants and is retained only as process-order evidence [86].

For hot-forming–quenching routes, solution treatment, transfer, deformation and die cooling must be distinguished. The AA6082 direct/indirect hot forming and quenching (HFQ) study compared mechanical response, microstructure and formed B-pillar sections; its conclusions identify lower formability and strength for the indirect route under the studied conditions, while shape accuracy and springback were not evaluated [87]. These are method and route comparisons in another alloy and not evidence of an Ω-alloy damage-tolerance window. Hot-forming reviews help identify temperature-dependent flow and thermal-history controls [11]. Target-material measurements are still needed to determine whether an Al–Cu–Mg–Ag sheet retains a suitable solute/precipitate state and crack resistance after such a route.

CAF combines imposed curvature, elevated-temperature deformation, and aging with cooling and unloading arranged according to the particular route. The example in Figure S3 cools under constraints before unloading; this order is not assumed for all CAF operations. Springback depends on the constitutive response, geometry, and loading arrangement [12,88]. In Figure S3, accumulated equivalent inelastic strain includes the illustrated plastic and creep contributions and is non-decreasing. Recoverable elastic strain is excluded; total strain and a signed stress component may decrease during unloading. A local stress component can remain nonzero after release because equilibrium applies to the spatial field. Pre-deformation studies in AA2219 offer useful constitutive-method examples for coupling creep, strength and precipitation [89], but their θ-dominated response is not Ω calibration for the target alloy.

The AGEFORM four-point-bending comparison provides direct AA2139 evidence [8]. Five identifiable markers in Figure 43 of Ref. [8], labeled AA2139-T851 by the source, span approximately 0.12–0.24% imposed maximum bending strain and 0.028–0.052% retained maximum bending strain. The source does not unambiguously assign the T851 label to a particular step of that forming sequence, so no additional temper sequence is inferred. Figure 5 replots these markers. Figure 44 of Ref. [8] is a separate strength–apparent-toughness comparison, not a paired retention test on these five specimens. For the separate clad AA2022-T8 alloy comparison, the authors reported no discernible R-curve difference between conventionally aged controls and inverse-age-formed sheets, using 760 mm wide L–T center-cracked tension (CCT) specimens. The sheets were formed on tools with radii of 1 m for 3.2 mm sheet and 2 m for 6 mm sheet and returned to a flat configuration before testing. These are tooling radii, not the curvature of the fracture-test panels. The result establishes a precedent for coupled forming and crack-resistance assessment without proving statistical equivalence. The 2008 journal version repeats this comparison in its Figure 25 and presents the related bending and strength–toughness results in Figures 40 and 41 [9]; these accounts are not independent replications.

Taken together, AGEFORM and the recent AA2139 experiment support monitoring both retained shape and the path-dependent material response. In Gautier et al., post-creep tensile tests showed roughly 45% higher yield stress and 10% higher ultimate tensile stress than T3, while elongation decreased; these changes contain aging and loading contributions [72]. The authors used the directional average (X_0_ + 2X_45_ + X_90_)/4, so the reported summary is not a simple mean of three orientations. A thermal-history-matched undeformed control is needed to isolate the loading contribution: it should follow the measured local temperature–time history as closely as practicable, with the same property measured in the control and loaded material. Other-alloy cases remain useful for evaluating specific methods: AA7475 section springback, Al–Li model/experiment deflection error, and buckling of stiffened panels are separate observables [90,91,92]. For route selection, the immediate task is to screen feasible shape and tensile outcomes together and then measure the damage endpoint on the retained candidates.

**Table 3:** Three thermomechanical interventions and their actual evidence.

Intervention	Initial State and Control	Timing/Variables	Microstructural Observation	Measured Performance	Scope and Limits
Pre-straining before aging [58]	3 mm AA2029; common solution/quench; 1.8/2.5/3.4% comparisons	513 °C/1.5 h → water quench → tensile prestrain → 175 °C at individual peaks	TEM-derived Ω volume fraction; boundary precipitates and competing phases	YS, elongation and IGC depth; 3–5 parallel tensile tests reported	Peak times covary; no zero-strain control in the plotted comparison; no fracture/FCG endpoint.
Loading during aging [68,72]	Ref. [68] same bicrystal with 0/50/100 MPa; Ref. [72] separate AA2139-T3 extracted from 120 mm plate	Ref. [68] 180 °C/53 h; 100 MPa yields. Ref. [72] 160 °C, 50/100/150 MPa, 0/45/90° to RD	Ref. [68] grain/near-boundary Ω fractions; Ref. [72] EBSD KAM response	Ref. [68] microscopy; Ref. [72] DIC creep and subsequent tension	Two target-family material forms; KAM is not a phase fraction. Loaded creep is not by itself a shaped-panel CAF demonstration; n per configuration NR.
Creep-age forming [8,90]	Ref. [8] AA2139-T851 source-labeled bending series; Ref. [90] separate pre-aged AA7475 stiffened panels	Ref. [8] imposed bending and post-forming strain. Ref. [90] 163 °C/24 h, 7 atm after 121 °C/6 h pre-aging	No paired Ω quantification for the plotted AA2139 bending markers	Ref. [8] five bending markers; Ref. [90] springback at five sections from mean deflections of three panels	AA2139 marker-specific thermal recipe/thickness/n NR; AA7475 is an other-alloy springback comparison. Neither plotted case validates formed-AA2139 fatigue or fracture retention.

Warm compression at 200 °C [73] is a timing probe, not CAF. HFQ in Figure S3 is a manufacturing route, not a surrogate for loading during aging.

**Figure 6:**
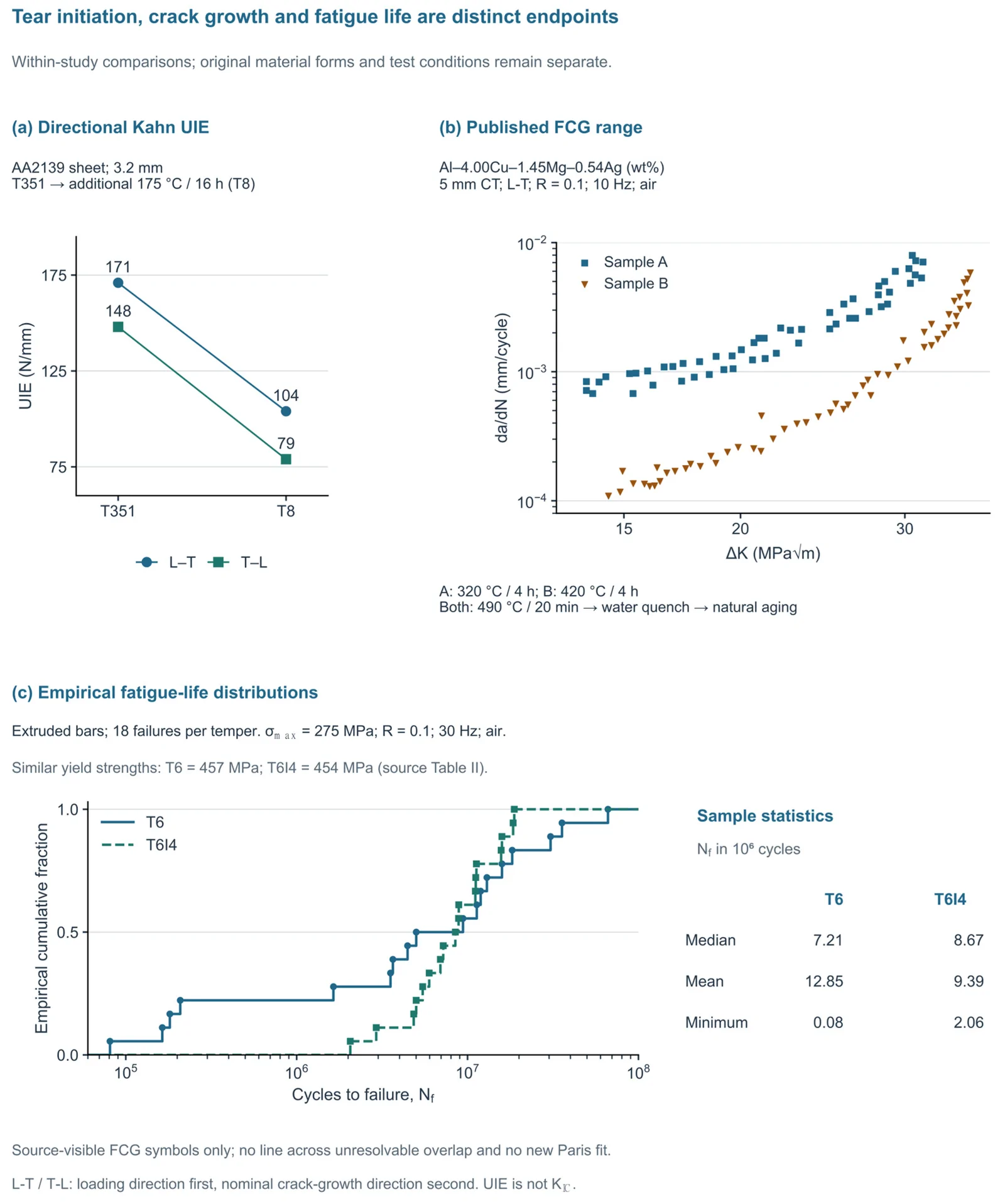
Three distinct damage-related endpoints are retained as within-study comparisons. (**a**) Kahn UIE for 3.2 mm AA2139 sheet in L–T/T–L [15] (source Table 3): 171/148 N/mm in T351 and 104/79 N/mm after the additional 175 °C/16 h treatment, designated as T8. Loading direction is listed first. The source reports at least two tests per condition/orientation; exact counts for Table 3’s values and uncertainty are not separately given. (**b**) FCG data digitized graphically from source Figure 2 of Ref. [16] and replotted on conventional log–log axes. The 5 mm Al–4.00Cu–1.45Mg–0.54Ag–0.52Mn CT specimens were tested in L–T at R = 0.1 and 10 Hz in room-temperature air. A/B received 320/420 °C for 4 h, then the common 490 °C/20 min treatment, water quench and natural aging (duration NR). The 54 points for A and 57 for B are extracted graphical coordinates and not counts of independent specimens; individual points do not necessarily represent separate specimens. Only identifiable published symbols are shown; unresolved overlaps were omitted, with no extrapolation or new Paris fit. The extraction and checking procedure is described in Methods S1. (**c**) Empirical cumulative distributions (i/n) of all 36 tabulated failures in extruded Al–Cu–Mg–Ag bars [93]: 18 per temper at σ_max_ = 275 MPa, R = 0.1 and 30 Hz in air, with no run-outs. After 525 °C/5.5 h and water quenching, T6 used 185 °C/10 h and T6I4 used 185 °C/2 h plus >1200 h natural aging. Medians, arithmetic means, and minima are distinct descriptors; a sample minimum is not a design lower bound. The panels do not define a pooled damage-tolerance ranking.

## 5. Directional Fracture, Fatigue and Environmental Response

Selected figure-driving and complementary damage studies are not a census of the manuscript bibliography. NR means not reported in the inspected source; any unexamined field must remain NE. Full composition and additional test metadata, including complementary long-crack and environmental-cracking comparisons, are provided in Table S1. Endpoint definitions are given in Table 1. Geometry rechecked in [93], PDF pp.2,4–5/Figure 4a; [94], PDF p.2/Figure 1; and [95], PDF pp.2–3/Figure 1. Source method names identify the reported protocols and do not assert independent standards-compliance certification. The studies discussed in this section address different stages of damage under distinct material and test conditions. Table 4 records these conditions and the limits that govern the following comparisons.

**Table 4:** Conditions of selected damage studies considered before cross-study synthesis. NR denotes unreported information in the inspected source, while NE denotes information not extracted or resolved for this comparison. WQ denotes water quenching.

Source/Endpoint	Material/Form	History and Geometry	Loading/Environment	Sample Count/Uncertainty	Permitted Interpretation
Ref. [31] Kahn UIE	Al-Cu-Mg-Ag, 1.6 mm sheet	Four Mg/thermal combinations; 2% pre-stretch, 160 °C peak age; L loading/T crack per schematic	Room temperature; 1 mm/min	Kahn n NR; error-bar definition NR; graph-read extents supplied	Tear-initiation energy per ligament area; not measured K_IC_
Ref. [15] directional UIE	AA2139, 3.2 mm sheet	T351 vs. additional 175 °C/16 h (T8); L–T and T–L	Quasi-static Kahn testing; endpoint from Source Table 3	At least two Kahn tests per state/orientation; exact Source Table 3 n and dispersion NR	Within-study directional tearing; neither life nor long-crack growth
Ref. [16] long-crack growth	Al-4.00Cu-1.45Mg-0.54Ag-0.52Mn; 5 mm plate	A 320 °C/4 h; B 420 °C/4 h; both 490 °C/20 min → WQ → natural age; compact-tension (CT) specimen, 45.6 × 38 × 5 mm; L–T	R = 0.1; 10 Hz; room-temperature air	Independent n NR; 54/57 graphically extracted coordinates, not independent specimens; reading bounds only	da/dN–ΔK within the published recoverable range; no threshold or new Paris fit
Ref. [93] fatigue life	Al-5.60Cu-0.45Mg-0.45Ag-0.30Mn-0.18Zr; extruded parent bars 70.6 × 27.6 mm	525 °C/5.5 h → WQ; T6 185 °C/10 h; T6I4 185 °C/2 h + >1200 h natural aging. Round dog bone: gauge length ≈ 12.7 mm, diameter ≈ 6.1 mm; longitudinal loading; low-stress ground surface	σ_max_ = 275 MPa; R = 0.1; 30 Hz; laboratory air	18 failures per temper, no run-outs; all 36 Source Table III values retained	Target-family bars: empirical total-life distribution; sample minima are not design allowables
Ref. [94] pre-corroded fatigue and fixed-cycle fitted stress	AA2139, 3.2 mm laser-welded sheet; T3/T8 before welding	4047 filler; 0/720 h salt spray (5 wt% NaCl, 35 ± 1 °C, pH 6.5–7.2). ASTM E466 [96] coupon: 194 mm overall, 14 mm reduced width, 18 mm grip width, transition radius 120 mm; 3.2 mm sheet; longitudinal loading	R = 0.1; 30 Hz; fatigue after exposure. The source does not specify the E466 edition. Current ASTM E466-21 [96] is cited as general methodological background only.	24 observations: 19 failures + 5 run-outs; one table/plot conflict preserved	Original fitted σ_max_ = 115/55 MPa at 10^7^ cycles; not an endurance limit or forming-retention ratio
Ref. [95] fracture-surface morphology	Additively manufactured (AM) EOS Al2139; vertical build; machined surface	14 mm diameter printed blank; machined round specimen: 90 mm overall, 15 mm gauge length, gauge diameter 6 ± 0.05 mm; T4 490 °C/45 min + 3 d natural aging; longitudinally polished; source Figure 8a,c	Axial fatigue; R = 0.1; 20 Hz; room air	Two selected source panels, not independent-replicate n; no new quantitative fatigue comparison	Target-family AM defect morphology only; not wrought-sheet forming or welded-corrosion validation [94]
Ref. [18] J–R-derived Kc	Al–Cu–Mg–Ag, 2.3 mm sheet	T8; 135 °C exposure for 0/1000/3000 h; L–T CT, W = 50.8 mm	Room-temperature J–R; ASTM E1152-87 [97]; Δa = 2 mm	Fracture n/uncertainty NR; >20 TEM negatives/state are not fracture replicates. ASTM E1152-87 [97] is the historical edition explicitly reported by the source.	90/74/75 MPa√m; 74 → 75 is not resolved recovery; not K_IC_
Ref. [98] low-cycle fatigue	Al–4.5Cu–0.56Mg–0.77Ag; specimens from ≈20 mm parent plate	T6 vs. T840 (40% cold rolling); 190 °C/2 h; round gauge Φ5 × 18 mm; L direction	Fully reversed tension–compression; constant total strain amplitude 0.4–1.0%; 0.5 Hz	Fatigue n per amplitude NR; ≥3 tensile tests do not define fatigue n	Cyclic stress–strain and 2N_f_ strain–life; shared T6/T840 lineage with [99]
Ref. [99] low-cycle fatigue	Same alloy family; ≈20 mm parent plate	520 °C/1 h → WQ; T83: 3% stretch → 190 °C/2 h; round gauge Φ5 × 12.5 mm; longitudinal	Symmetric tension–compression; constant total strain amplitude 0.4–1.5%; 0.5 Hz	Fatigue n per amplitude NR; ≥3 tensile tests reported separately	T83 compared with earlier T6/T840 baselines; reused data are not new independent tests
Ref. [40] FCG and corrosion	Cu 3.19/3.51/3.78 wt%; 2.5 mm crack-specimen thickness	Separate peak-aged states; 50 × 48 × 2.5 mm (source Figure 1c)	Precracking: ≈2 mm at Kmax = 10 MPa√m; FCG: source-reported cyclic load-control program; R and frequency NR; separate 6 h IGC	Independent fatigue n NR; precipitate and hardness counts are not fatigue n	Qualitative multi-endpoint comparison; no pooled growth curve. Current ASTM E647-24 [100] is general methodological background only; the source does not specify the edition used.

### 5.1. Tear Resistance and the Limits of a Strength-Based Ranking

The AA2139 tear experiment in Figure 6a demonstrates that the final aging state can change both the magnitude and directionality of fracture-related response [15]. For 3.2 mm sheet, Kahn unit initiation energy decreased from 171 to 104 N/mm in L–T and from 148 to 79 N/mm in T–L when comparing T351 with the reported T8 treatment at 175 °C for 16 h. The first letter denotes loading direction and the second crack-growth direction, with L parallel and T transverse to rolling. The result is a temper- and orientation-dependent tear response in the specified geometry. It is not a general conversion between tensile strength and K_IC_ nor a controlled test of forming alone.

This distinction has a microstructural basis. Particle cracking or decohesion, void growth, strain localization and the orientation of particle arrays can influence ductile tearing differently from the resistance to initial yielding. The RX226 plate investigation found comparatively weak tensile-strength anisotropy while retaining orientation- and position-dependent fracture characteristics [5]. The AA2139 tear study similarly connects the response with the material microstructure and damage development [15]. For a formed skin, a stronger local state can therefore coexist with less favorable resistance to crack initiation or tearing. The controlling question is which damage mechanism is activated under the relevant constraint, rather than whether a single tensile indicator increased.

For Al–Cu–Mg–Ag sheet, Haynes and Gangloff characterized initiation and stable-tearing resistance under monotonic loading in 3.2 mm AA2519 with Mg/Ag additions and a 2.3 mm lower-Cu variant (C416), using L–T compact-tension specimens and J-integral resistance curves [101]. The full text’s methods and results distinguish initiation from subsequent plane-stress tearing over ambient and elevated temperatures. Fractography links the response to constituent particles, void growth and void-sheet coalescence; the micromechanical model additionally requires an adjustable critical-distance parameter. The different temperature dependencies of initiation and stable-tearing resistance show why a tensile or precipitate descriptor alone cannot rank both endpoints. Fracture during elevated-temperature loading and room-temperature fracture after a prior thermal exposure also remain separate comparisons.

Three-dimensional observations add a direct link between local structure and tearing. Tomography of AA2139 tear specimens showed directionally organized pores and void linkage consistent with toughness anisotropy [102]. In situ laminography then followed void growth and coalescence during monotonic crack extension in laterally extended sheet [103]. Flat-to-slant transition studies identified a change from high-triaxiality void growth to shear-dominated coalescence [104]. Combined laminography and digital volume correlation (DVC) resolved slant strain bands before major void growth; the corresponding damage-model simulation reproduced slant fracture more successfully than the early strain field [105]. These linked studies provide complementary observations within a related experimental lineage. They are not four independent demonstrations of fatigue improvement after forming.

Boundary and particle evidence also limits a single-descriptor explanation. Li and Reynolds examined 2.3 mm Al–Cu–Mg–Ag sheet in T8 and after 1000 or 3000 h at 135 °C [18]. YS was 498/494/465 MPa and the reported Kc was 90/74/75 MPa√m. Kc was calculated as √(JE) at 2 mm crack extension, rather than measured K_IC_. Because fracture-test uncertainty was not reported, the 74-to-75 MPa√m difference is not interpreted as a resolved recovery. PFZ width and grain-boundary precipitate characteristics changed together, so these states do not isolate the PFZ contribution. The lower J–R-derived Kc at the sampled 1000 h condition, with little change in YS, demonstrates the need for a stable-tearing endpoint; the paper’s grain-boundary fracture strain is model derived.

Temperature-dependent void-sheeting experiments used 3.2 mm AA2519 sheet modified with Mg and Ag in the T87 condition [106]. Fracture surfaces produced at 25, 150 and 175 °C, with notched-bar observations at 25 and 150 °C, linked the higher-temperature ductility to reduced dispersoid-assisted secondary void nucleation. A separate AA6156 quench study directly observed boundary-associated failure and different sensitivities of tear initiation and propagation energies [107]. The latter is useful as a measurement and mechanism comparator, not as target-alloy validation. Historical AA2139 development also distinguished Mn- and Zr-bearing dispersoid populations [108]. Taken together, the sources support examining constituent particles, dispersoids, grain-boundary precipitates and adjacent PFZs, and strain localization alongside strengthening precipitates. They do not establish that a particular PFZ width or Ω fraction universally maximizes fracture resistance.

### 5.2. Texture, Crack Paths and Crack Closure

Figure 6b uses a separate Al–4.00Cu–1.45Mg–0.54Ag (wt%) study to examine fatigue crack growth [16]. Both conditions were naturally aged, but the preceding anneals were 320 °C for 4 h for A and 420 °C for 4 h for B. The B condition contained more pronounced P and Goss texture, together with other microstructural changes. Tests used 5 mm compact-tension specimens in L–T in room-temperature air at a load ratio of 0.1 and a frequency of 10 Hz. These conditions differ from the AA2139 tear experiment in composition, treatment, thickness, geometry and endpoint. Keeping the datasets separate is necessary before attempting any mechanistic synthesis.

At ΔK = 15 MPa√m, the source text reports A/B rates of 8.15 × 10^−4^/1.54 × 10^−4^ mm/cycle; at 30 MPa√m the rates are 6.25 × 10^−3^/1.59 × 10^−3^ mm/cycle [16]. These four textual values are cross-checks; Figure 6b is based on separately digitized visible markers. Figure 6b uses 54 A and 57 B coordinates digitized graphically from identifiable published symbols in source Figure 2 and replotted on ordinary log–log axes; these coordinates do not establish the number of independent specimens. Overlap limits the recoverable subset; neither hidden symbols nor threshold/instability ranges are reconstructed, and no new Paris fit is introduced. The four textual anchors were retained for checking without moving extracted points to force agreement. The source’s crack-deflection and closure explanation is considered alongside the actual EBSD paths in Figure 4g,h. Its closure estimates of 0.25 and 0.73 are analytical; they are not measured opening loads. The shared material is Al-4.00Cu-1.45Mg-0.54Ag-0.52Mn, with A/B anneals followed by the common 490 °C/20 min treatment, water quenching, and natural aging; it is not relabeled AA2139.

Recent work on naturally aged Al–Cu–Mg–(Ag) alloys has further shown that Cu/Mg ratio and Ag addition alter Goss-grain populations, coarse particles, and crack-growth behavior altogether [109]. Reviews of texture engineering place such observations within a wider range of aluminum processing routes [110]. The transferable concept is that neighboring-grain orientations can modify slip transfer and crack deflection. The non-transferable shortcut is to assign a fixed crack-growth benefit to a texture component independently of grain size, particle distribution, temper and load ratio. Texture should be treated as a spatially organized part of the microstructure, rather than only as an orientation-distribution-function peak intensity.

### 5.3. Connect Aging Pathways to Fatigue Without Collapsing the Test Conditions

Bai and co-workers linked aging path to fatigue crack propagation in a low-Cu/Mg Al–Cu–Mg–Ag alloy [111]. The Ag-containing material was 2 mm sheet; compact-tension (CT) specimens were tested in the L–T orientation at R = 0.1 and 10 Hz in laboratory air. Initial aging at 170 °C for 0.5 h improved crack-propagation resistance relative to the T351 route in the under-aged conditions studied, whereas subsequent exposure at 150 °C for 500 h reduced resistance. The authors associated the remaining difference with Ω-dominated versus S(S′)-dominated microstructures. This connects treatment sequence to a crack-growth endpoint, complementing the tensile evidence in Figure 3. The comparison is retained qualitatively; no new growth-rate curve or universal pre-aging optimum is inferred.

Burba et al. show why a central fatigue-life value is insufficient [93]. The target-family Al–5.60Cu–0.45Mg–0.45Ag developmental alloy was supplied as extruded bars, not rolled sheet. T6 (185 °C/10 h) and T6I4 (185 °C/2 h followed by more than 1200 h of natural aging) gave similar yield strengths, 457 and 454 MPa. At a maximum stress of 275 MPa, R = 0.1 and 30 Hz in laboratory air, 18 specimens per state were tested. Replotting every lifetime in Table III of Ref. [93] gives sample medians of 7.21 × 10^6^ and 8.67 × 10^6^ cycles, but sample minima of 80,823 and 2,059,268 cycles (Figure 6c). These are sample descriptors, not design lower bounds. The source calls the 50% cumulative-distribution position a mean. Here, the sample median is calculated explicitly from the ninth and tenth ordered lifetimes, rather than read from the logarithmic plot or identified with one empirical cumulative distribution function (ECDF) step. Arithmetic means are 12.85 × 10^6^ and 9.39 × 10^6^ cycles, respectively; the ordering of these means differs from that of the medians.

The same study separated initiation from small-crack propagation using fractography and focused-ion-beam (FIB)-notched specimens. The four short-life T6 specimens fell within an author-calculated crack-growth-life envelope, whereas small-crack growth rates were largely similar between aging treatments at room temperature and at 130 °C under the separately specified dwell cycle [93]. The authors interpreted the lower tail in terms of local initiation susceptibility, including particles and the surrounding grain structure. Independent AA2139 T6/T840 work reported increased tensile strength after a route including 40% rolling without a comparable fatigue-life increase [112]. Its full text describes plate-derived specimens tested at 50 Hz and R = 0.1; that comparison is retained qualitatively here. Together with the tear and long-crack studies, these observations support screening by damage stage: retain the strength/ductility envelope, examine initiation sites and life dispersion, and measure small- and long-crack growth under their corresponding conditions. A tensile improvement supports candidate selection but does not predict fatigue performance.

A 2026 SSRN preprint directly examines Al–Cu–Mg–Ag peak-aged material after 1%, 2%, and 4% pre-stretching and includes fatigue-crack-propagation comparisons [113]. It is relevant to the missing process–crack link and precludes describing this topic as wholly unexplored. No corresponding peer-reviewed article was identified through the title/author searches used here. Its reported relationships are therefore kept outside the quantitative main figures and are not used to establish an optimum prestrain.

Other-alloy studies help specify the controls needed to examine this link. Prestrained AA2524-T3 investigations varied in terms of prestrain direction, crack orientation, and load ratio, showing that these variables belong in the same experimental description [114,115]. A recent Al–Cu–Li–Sc study likewise examined prestrain and fatigue-crack-growth resistance, but its distinct chemistry limits direct transfer to Ω-strengthened sheet [116]. For Al–Cu–Mg–Ag forming, a comparison should measure the crack-growth curve over an identified regime, retain orientation and stress ratio, and characterize the state adjacent to the tested crack path. A single rate at one ΔK can screen states; it cannot by itself determine an inspection interval.

Strain-controlled low-cycle fatigue adds another endpoint rather than another set of Figure 6c lifetimes. Gazizov and Kaibyshev compared T6 with a T840 route containing 40% rolling and measured cyclic stress–strain response and strain–life behavior [98]. A later study examined 3% tensile prestrain followed by 190 °C peak aging (T83), using earlier T6/T840 results as comparisons [99]. These publications share a comparison lineage; reused baselines do not constitute additional independent tests. Their reversal-based strain–life representation (2N_f_) must also be distinguished from cycles to failure (N_f_). Since the strain amplitude, cyclic constitutive response, and product history differed from the stress-controlled extruded-bar experiment, no combined life distribution or universal ranking of T6, T83, T840 and T6I4 is constructed. The useful connection is that a route selected by monotonic strength can change cyclic plasticity differently, which requires a test at the intended cyclic regime.

### 5.4. Grain-Boundary Chemistry and Environmental Damage

Grain-boundary morphology alone is an incomplete predictor of environmental cracking. Scully and co-workers examined two Al–Cu–Mg–Ag alloys and developed an electrochemical interpretation of aging-dependent intergranular stress-corrosion susceptibility [117]. Their results relate susceptibility to the difference in Cu concentration between the grain interior and boundary-adjacent material, rather than simply to the presence of a particular boundary phase. This gives a stronger basis for interpreting forming-induced changes than a rule such as “more discontinuous precipitates always improve corrosion resistance.” Precipitate continuity, solute depletion, local electrochemical response, and the loading condition need to be considered together.

The distinction between corrosion modes is equally important. Comparative work on 2xxx alloys shows that localized-corrosion rankings depend on the alloy microstructure and exposure protocol [118], while a recent aviation-alloy review emphasizes the additional interaction with cyclic loading [119]. The lower immersion-corrosion depths after prestrain in Figure 3 are therefore useful evidence about that specific exposure, not proof of improved corrosion-fatigue life. A forming-retention study should connect the observed corrosion site to the actual crack origin and distinguish pre-corrosion followed by fatigue from simultaneous environmental exposure and cycling. Otherwise, changes in initiation and long-crack propagation remain conflated.

The Cu-content study of Ouyang et al. provides a more direct multi-endpoint comparison within one alloy family [40]. Measured Cu contents of 3.19, 3.51, and 3.78 wt% were accompanied by small Mg/Ag differences; the study evaluated tensile properties, ASTM E647-type crack propagation [100], immersion IGC and electrochemical response. Higher strength did not coincide with the most favorable crack-propagation or corrosion response. Its explanation combines intragranular Ω/S′ changes with boundary S/θ distributions and electrochemistry. These associations strengthen the case for multi-endpoint screening, but the individual contributions are not isolated causal effects. The 2.5 mm specimen thickness is identified in source Figure 1c, while the load ratio, cyclic frequency, and independent FCG replicate count are not reported in the inspected methods; these omissions remain in Table 4, so its crack-growth results are used qualitatively rather than pooled with Figure 6b. Neither its immersion protocol nor the pre-corroded welded-sheet test in Section 6.3 represents simultaneous corrosion fatigue.

## 6. Subsequent Manufacturing and Fatigue- and Fracture-Relevant Local States

### 6.1. Residual Stress Is a Field Inherited and Modified by Processing

**Figure 7:**
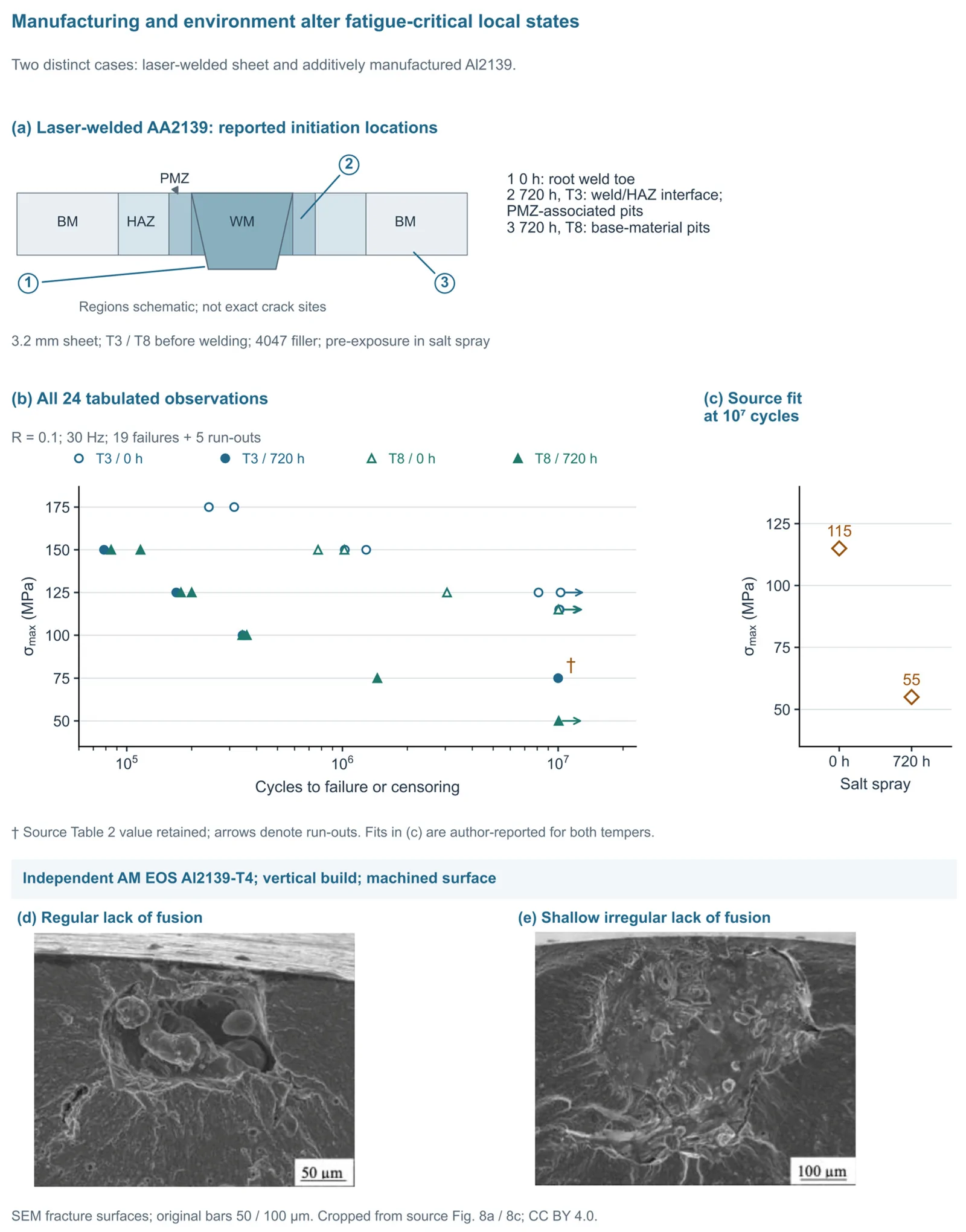
Manufacturing and environmental effects in two separate Al2139 product contexts. (**a**–**c**) Laser-welded 3.2 mm AA2139 sheet, with T3/T8 denoting the pre-weld temper [94], using 4047 filler, 3 kW laser power, and 1.8 m/min travel speed. (**a**) Original non-scale schematic of base material (BM), HAZ, partially melted zone (PMZ), and weld metal (WM). Leaders identify reported initiation regions: the unexposed root weld toe, exposed-T3 weld/HAZ-interface neighborhood with PMZ-associated pits, and exposed-T8 base-material pits. They are not measured point coordinates. (**b**) All 24 tabulated observations at R = 0.1 and 30 Hz along RD: 19 failures and five right-censored run-outs, after 0/720 h salt-spray exposure. Right arrows mark run-outs. The dagger retains the T3/720 h/75 MPa value of 10,002,000 cycles despite the source table/plot disagreement. (**c**) Author-fitted maximum stresses of 115/55 MPa at 10^7^ cycles for 0/720 h, reported for both tempers; no new S–N fit or endurance limit is inferred. (**d**,**e**) Separate SEM examples of regular/shallow irregular lack of fusion in vertically built, machined EOS Al2139-T4 [95] (original Figure 8a,c); 490 °C/45 min plus three days of natural aging, axial fatigue at R = 0.1 and 20 Hz in room air. These are different defect examples, not images of the welded or pre-corroded specimens. Their use is qualitative because source fatigue-count and stress-definition inconsistencies remain unresolved. Scale bars: 50/100 µm. Image credit: Kumarasinghe et al. [95], Journal of Materials Research and Technology 44 (2026) 6048–6062, DOI:10.1016/j.jmrt.2026.09.055, © 2026 the authors, CC BY 4.0; rectangular crops and proportional placement only. No photographs from [94] are reproduced.

Subsequent trimming, local thinning, machining, joining and surface treatment act on the state produced by forming. Material removal redistributes a pre-existing stress field; mechanical cutting and joining can additionally introduce local plasticity, thermal gradients and new crack origins [13,14]. A useful control therefore links each operation to a mapped material location and to the observable it can change: distortion, surface condition, local hardness, corrosion susceptibility, or crack behavior. Figure 7 develops the direct AA2139 welded-sheet example.

For fracture assessment, the stress sign without coordinates and a location is insufficient. A useful description specifies the tensor component, depth, reference surface and spatial variation relative to the crack. Under the linear-elastic mode-I superposition, the total stress-intensity factor can be written as K_total_ = K_applied_ + K_res_. A static residual contribution may leave the nominal applied ΔK unchanged while altering the effective load ratio, opening behavior and maximum stress-intensity factor; K_res_ can also evolve as the crack advances or the field relaxes. Lammi and Lados demonstrated a combined ΔK–K_max_ interpretation with closure and residual-stress corrections in AA6061-T61 [120]. This is methodological evidence, not a calibrated crack-growth model for Al–Cu–Mg–Ag.

### 6.2. Machining: Separate Distortion Control from Fracture Benefit

Machining studies provide useful controls for limiting geometrical distortion. Quasi-symmetric material removal in AA7075 reduced distortion relative to the one-sided route studied [121]. Work on AA7050-T7451 pockets examined how initial residual stress and the toolpath influence distortion [122]. Such findings support recording blank location, removal sequence and support conditions when comparing manufactured specimens. They do not establish that the least distorted part has the best fatigue performance. A low global distortion can coexist with a locally unfavorable stress, roughness or defect population near a crack origin.

### 6.3. Joining, Surface Treatment and Environment-Sensitive Initiation

Kermanidis et al. [94] tested laser-welded 3.2 mm AA2139 sheets with T3 or T8 conditions, as established before welding using 4047 filler. The fatigue tests were longitudinal at R = 0.1 and 30 Hz after either no salt-spray exposure or 720 h of exposure. The source reports unexposed initiation at the root weld toe. For exposed T3 specimens, it describes initiation at the weld-metal/heat-affected-zone (HAZ) interface and pits associated with the partially melted zone (PMZ); exposed T8 specimens initiated at base-material pits. Figure 7a maps these reported regional categories without inventing point coordinates or treating subsequent passage through the PMZ as proof of a universal PMZ origin. Figure 7b preserves all 24 tabulated observations, including five run-outs and the flagged T3/720 h/75 MPa entry of 10,002,000 cycles that conflicts with the source plot. Figure 7c retains the authors’ 115 and 55 MPa fitted maximum stresses at 10^7^ cycles. These are finite-life fit outputs, not a new endurance limit or a forming-retention measurement.

The source authors interpret the exposure effect in terms of local pits acting as stress concentrators and shortening the initiation stage. They associate the T3 response with coalesced pits in the PMZ neighborhood and the T8 response with pits in the base material, where the boundary state differs from that produced in the heated weld neighborhood [94] (source Section 3.3). This is the source’s mechanistic interpretation; the present review does not add measurements of local potential, PFZ chemistry, local stress intensity or initiation duration. The practical synthesis is to preserve the spatial comparison: map the weld toe, interface/PMZ neighborhood and base material before and after the actual exposure, and retain the same loading and endpoint definitions. Similar fitted fatigue stresses can otherwise hide a change in the region that requires protection or inspection. The experiment concerns fatigue after pre-exposure, and coupon preparation does not reproduce the complete intact-panel residual-stress field.

Figure 7d,e adds a separate target-family example of manufacturing defects from vertically built, machined EOS Al2139-T4 tested in room-temperature air at R = 0.1 and 20 Hz [95]. Its selected scanning electron microscopy (SEM) fracture surfaces show regular and shallow irregular lack-of-fusion defects in different examples; they are not before/after views of one specimen or micrographs of the welded or pre-corroded material in Figure 7a–c. The AM study is used qualitatively because sample-count and stress-range/amplitude inconsistencies remain in its record. It motivates recording the lack-of-fusion defects after the chosen manufacturing route, but it supplies no quantitative extension of the welded-sheet corrosion data or validation of rolled-sheet forming retention.

The resulting manufacturing control is spatial: verify the base material, weld toe, partially melted region and heat-affected region after the chosen temper and exposure, rather than inspecting only the visibly modified weld center. Other-alloy evidence supplies complementary methods. In AA2024-T3 refill friction stir spot welded (RFSSW) lap joints, laser shock peening was associated with author-fitted fatigue-strength factors of 1.51 and 2.82 at 2 × 10^6^ cycles, while strong three-dimensional gradients limited hole-drilling stresses to qualitative interpretation [123]. These are strength factors at a fixed life and are not life multipliers. Non-through-crack experiments further show why crack geometry belongs in the comparison [124]. The common assessment should therefore include the actual initiation location and geometry, together with a stress field of appropriate spatial resolution.

## 7. Discussion: Evidence Integration and Conditional Transfer to Curved Panels

### 7.1. Conditional Synthesis Across Composition, Sequence and Damage

Three comparisons clarify how processing should be evaluated. First, strengthening descriptors and damage resistance respond to different parts of the material state. In the AA2029 prestrain series, YS increased from 428 to 459 MPa while the reported Ω volume fraction decreased from 1.44% to 0.96% [58]. In the separate thermal-exposure study, J–R-derived Kc fell from 90 to 74 MPa√m after 1000 h at 135 °C while YS changed from 498 to 494 MPa [18]. The first comparison shows that Ω volume fraction alone does not account for the measured yield-strength trend. Competing precipitates and deformation-induced dislocations are plausible additional contributions under those conditions, but their relative contributions were not isolated. The second comparison shows that largely retained yield strength can coexist with lower resistance to stable tearing, represented by J–R-derived Kc at 2 mm crack extension. A useful evaluation therefore combines intragranular precipitation with boundary and particle observations and a specified damage test. Neither comparison isolates a universal Ω- or PFZ-controlled fracture law.

Second, the state entering deformation and the time at which deformation occurs are controllable variables. Small-stretch APT studies observed reduced Mg–Ag co-clustering under their own conditions [61,62], whereas inserting 5% compression later in a 200 °C hold changed hardness non-monotonically [73]. Qin et al. further found that pre-aging before 5% stretching gave a different precipitate and tensile response from direct stretching [74]. Together, these results make quench-to-deformation storage and pre-aging part of the comparison, instead of these being incidental handling details. They support matched timing experiments within one material pedigree; differences in alloy, deformation mode, and aging endpoint prevent a common numerical optimum from being inferred.

Third, manufacturing can change the location that controls fatigue without that change being apparent in a global fitted endpoint. After salt-spray pre-exposure, the welded-AA2139 study associated T3 initiation with the weld/HAZ-interface neighborhood and T8 initiation with base-material pits, although it reported the same 115-to-55 MPa fitted stress change at 10^7^ cycles for both tempers [94]. The separate AM observations identify lack-of-fusion defects as another manufacturing-specific origin [95]. Evaluation should therefore follow the susceptible region through the complete route and exposure, alongside the absolute fatigue response. AM fracture surfaces inform defect assessment but do not establish the corrosion mechanism of a welded sheet.

Table 5 translates these findings into conditional controls and the comparisons needed to test them. AGEFORM already established coupled forming–property assessment; the remaining target-specific connection is a mapped local history in Ag-bearing sheet, its post-aging microstructure, and a matched crack endpoint, with specimen extraction and subsequent manufacturing accounted for [8,15,72,94].

Supplementary Table S1 organizes the selected figure-driving sources by material form, intervention, observable, and interpretation. It is a selected-source register rather than a measure of coverage of the complete bibliography. The 2006 and 2008 AGEFORM accounts are related versions; selected Mg/homogenization tensile values have unresolved specimen linkage; and several FAA reports describe one program. The source-level entries make those dependencies visible and identify which conclusions remain useful despite incomplete uncertainty or access information.

### 7.2. Matched Controls for a Forming-Retention Experiment

A primary comparison starts with the same material pedigree and evaluates formed and unformed material after matched final heat-treatment schedules. Pedigree includes the melt or batch, rolling and homogenization history, thickness position and initial orientation. Matching a final schedule does not force the final microstructures to be identical: those differences are part of the process response being investigated. It does prevent the simplest confounding comparison between a soft W-state starting sheet and a stronger T8 finished sheet. An undeformed control should be subjected, as closely as practicable, to the measured local temperature–time history of the formed material. Any remaining thermal mismatch should be documented, and the same property should be measured in the control and the formed material.

Parts A and B of Table 6 outline complementary contrasts for identifying thermal, deformation, and manufacturing effects. A common final heat treatment is one mechanism-identification strategy, though it is not a requirement that every optimized route use the same aging schedule. Route optimization may select a different final aging treatment for each forming path; it should then compare complete routes against a shared performance specification and report both achieved shape and absolute material properties. In either design, specimens should be linked to mapped local temperature, strain and thickness histories, and microscopy should sample the same regions as the mechanical endpoints. Replication is needed at the specimen and material level, in addition to multiple micrographs. Table 6’s parts A and B summarize the distinction between mechanism-identification contrasts and whole-route comparisons; Table S3 supplies a recording template.

Review synthesis. Applicable stages: initial material → after forming/aging → after subsequent manufacturing → structural validation. Matching histories does not require identical resulting microstructures.

Five contrasts interpreted using the two purposes in Table 6A. This is a proposed reporting framework and not an experimental result or validated standard.

For an endpoint Q whose larger value is favorable, a descriptive retention ratio can be written as R_Q_(x) = Q_formed_(x)/Q_control_, where x is the mapped location on the formed component associated with the specimen, and Q_control_ is the corresponding reference endpoint under matched conditions. For fatigue crack growth, the more informative ratio is R_g_(ΔK, R, environment) = g_formed_/g_control_, where R is the cyclic load ratio and g = da/dN, with the formed and control values evaluated at the same ΔK, R, and environment, for which a value below unity denotes a lower rate. These ratios are proposed reporting devices, not new constitutive laws or universal acceptance criteria. They should be accompanied by the underlying values, uncertainty, specimen counts and condition metadata. Combining them into one weighted damage-tolerance score would obscure the different physical meanings and is not supported by the heterogeneous evidence reviewed here.

Retention and suitability answer different questions. A ratio near unity preserves the reference response, but a reference below the required absolute property does not become suitable through retention. Accordingly, each comparison should display Q_formed_ and Q_control_, or both crack-growth curves, alongside the ratio and the application-specific requirement when one is available. The review proposes no universal pass value. Material-history matching estimates a defined intervention; whole-route comparison identifies the more useful process under a common design objective.

Specimen extraction introduces an additional boundary. Cutting a coupon from a curved region can relax residual stress, change constraint and remove thickness or surface features that contributed to the original structural response. Consequently, an extracted specimen may characterize a processed microstructure without preserving the complete structural stress state. Coupon results should therefore be distinguished from the response of the intact component. Paired measurements, stress-field reconstruction and appropriately validated models can then determine what is transferable. The sampled position and any straightening, machining or surface preparation must remain in the specimen history.

### 7.3. Retrospective Application to the AA2139 Weld Study

The AA2139 weld experiment offers a concrete application without combining unrelated datasets [94]. Its contrast is a pre-corroded versus uncorroded welded coupon within a specified pre-weld temper. The available absolute endpoint is the author-fitted maximum stress at 10^7^ cycles, changing from 115 to 55 MPa. The corresponding exposure ratio is 55/115 = 0.48, and the absolute decrease is 60 MPa. This calculation describes the penalty of the reported exposure under the coupon conditions; it is not a forming-retention ratio and does not turn a fitted quantity into an experimentally measured endurance limit. The scatter plot retains the tabulated observations and run-outs separately from that fitted endpoint.

The ratio alone would suggest a similar exposure penalty for T3 and T8, whereas the observed origin shift identifies locations for follow-up inspection assessment: the welded/adjacent region is critical in T3, and the base material becomes critical in T8 after the specified salt spray. To evaluate a forming effect, the minimum added contrast is a formed and an unformed set from the same sheet pedigree, taken through matched welding, aging and exposure histories, with equivalent sampling and test geometry. To evaluate intact-panel performance, a further comparison must retain or reconstruct the weld/forming residual field because coupon extraction relaxes it. A route-optimization study could instead vary the final age deliberately, provided it compares absolute requirements and identifies the complete route being optimized. This example identifies decisions supported by the current evidence and the measurements needed to extend them. This proposed forming contrast holds exposure fixed; it does not estimate a forming–exposure interaction.

### 7.4. Models Must Connect and Validate Distinct Observables

A useful model hierarchy connects forming fields, microstructural evolution and fracture response while preserving their different validation requirements. Texture-informed plasticity can help estimate local strain and stress, but agreement with a forming shape does not validate precipitate kinetics [51,52]. A precipitation-strengthening model can reproduce hardness or yield strength without validating crack growth [30,67]. Conversely, an anisotropic phase-field study calibrated to AA2024-T351 reproduced aspects of crack path and growth rate but identified limitations associated with two-dimensional treatment and thickness-dependent constraint [125]. Each agreement supports the observable and range actually tested, rather than the entire process-to-failure chain.

The priority is to connect models through experimental constraints. Forming measurements should constrain local histories; microscopy should constrain state evolution; fracture or fatigue tests should constrain the damage law. Parameters inferred from one dataset should be distinguished from tests used for prediction assessment, particularly when several papers share specimens, methods or numerical summaries. Where full validation is not feasible, sensitivity studies can identify which uncertain state variables most affect the predicted ranking. A transparent range of predictions is more useful than a precise numerical process window unsupported by target-alloy fracture measurements.

### 7.5. Curved-Panel Evidence and the Specific Transfer Still Needed

Curved-panel damage-tolerance testing already provides an important structural precedent. The FAA emerging-metallic-structures program developed comparable fuselage-panel loading and examined crack growth and residual strength [126,127,128]. Its baseline Panel 1 used AA2524-T3 skin, whereas Panels 2 and 3 used AA2060-T8 Al–Li and AA2029-T3 clad aluminum skins, respectively, with Al–Li stringers and frames in both advanced panels. Differences in manufactured skin-pocket thickness required attention to stress-intensity matching rather than simply applying the same nominal load. These related documents are evidence from one program, not three independent demonstrations. The AA2029-T3 skin is target-family structural evidence within a mixed-material panel; the other skins provide structural comparison cases. These panel tests do not isolate retention after a matched Al–Cu–Mg–Ag forming route.

A separate European study describes testing and validation at the stiffened-panel level for fuselage concepts [129]. Together with the FAA evidence, it demonstrates that the final validation problem includes boundary conditions, load introduction, curvature and crack configuration. The remaining target-specific task is to place a characterized Al–Cu–Mg–Ag forming route within such an evidence chain. The intermediate curved element is especially useful: it can retain a representative local process history and constraint while permitting closer measurement than a full fuselage section. The appropriate scale should follow the claim being tested and the unresolved mechanism, rather than a fixed progression defined only by specimen size.

The seven transfer variables are curvature, thickness, constraint, residual stress, crack geometry, boundary conditions and manufacturing sequence. Curvature and restraint alter local multiaxial loading; thickness and crack geometry alter constraint and driving force; residual stress can relax during extraction and cycling; and the manufacturing order changes the local material and surface state. These variables must be measured, reproduced or bounded in the structural comparison. Table S4 provides a variable-by-variable recording and validation guide.

### 7.6. Methodological Limits of the Synthesis

The physical transfer limits above differ from limits of the review method. The recorded search batches are reproducible discovery records, but they are not a complete bibliographic census.

Measurement definitions, reporting omissions and within-source inconsistencies remain explicit. Graphical extraction recovers a published representation with reading uncertainty, not raw laboratory data. The resulting conclusions are conditional syntheses and proposed comparison designs. The journal’s standing or paper’s publication date did not replace appraisal of relevance, experimental conditions and the actual claim supported. Recent evidence is used alongside classic mechanism studies where each has a distinct function.

## 8. Conclusions

- O1—Composition and inherited states. Cu, Mg, and Ag alter precursor kinetics, competing precipitates, grain-boundary precipitates and adjacent PFZs, while homogenization and rolling determine the state on which forming acts. Comparisons should therefore select complete composition–history states and report precipitate number density, dimensions, volume fraction and spatial distribution separately. Cross-over alloys illustrate alternative forming–hardening strategies, but their T-phase, cluster, or η′ evidence does not establish Ω-alloy crack resistance.
- O2—Deformation and timing. Small tensile prestrain, rolling, loading during aging, and warm compression are distinct interventions. Their responses depend on the state when loading begins, local plasticity, orientation, and the measured quantity. The defensible control is to document these variables and test matched alternatives; the available evidence does not establish a universal optimum prestrain, stress or compression time for aircraft-skin damage tolerance.
- O3—Damage endpoints. Strength, tear initiation, J–R resistance, long-crack growth, cyclic plasticity, and total fatigue life require separate comparisons. Boundary-sensitive fracture, directional void linkage, and fatigue initiation can change without a corresponding tensile ranking. The selected life distributions, crack paths and pre-corroded weld results therefore support damage-stage-specific assessment, rather than a single strength-derived damage-tolerance score.
- O4—Controls and transfer. Building on AGEFORM and other coupled forming–property studies, PRDT records the intervention, reference, local state and endpoint, with absolute performance retained alongside descriptive ratios. Mechanism identification uses matched controls; route optimization can use different schedules under common requirements. Transfer to curved skins additionally requires curvature, thickness, constraint, residual stress, crack geometry, boundary conditions and manufacturing sequence. These are proposed reporting and validation requirements, not a newly validated process window.

## 9. Future Directions

The first priority is a matched comparison of prestrain before aging and loading during aging in one sheet pedigree. The key question is whether the different Ω/competing-phase and boundary states persist after the same final schedule and produce distinguishable crack-growth or fracture responses. No resolved difference in crack response, within the tested range and measurement uncertainty, would constrain the predictive value of the selected descriptors; it would not establish that they are mechanistically irrelevant. The second priority is a directional comparison across mapped high- and low-strain regions, with texture, particle distributions and crack paths assessed together. This tests whether a local forming benefit remains favorable in both relevant crack orientations.

The third priority is to distinguish microstructural retention from the contribution of a residual-stress field by comparing extracted coupons with intact curved elements and by documenting stress relaxation during preparation and cycling. The fourth is to connect grain-boundary and surface changes with the actual environmental crack origin, using exposure and loading conditions appropriate to the intended claim. Across all four priorities, the decisive improvement is a shared specimen lineage linking process history, microscopy and crack response. More strength data or additional schematic arrows alone will not resolve that connection. Conversely, a limited number of well-matched experiments can substantially improve the transferability of a much larger existing literature.

## Figures and Tables

**Table 5:** Cross-study synthesis translated into conditional controls. Each row combines a scientific question with the supported action and missing discriminating comparison; these are review proposals and not qualified process windows.

Question	Evidence Brought Together	Conditional Action	Interpretive Boundary	Next Discriminating Comparison
Composition and inheritance	More initial clusters do not uniquely determine Ω; SH/TH gives more numerous, smaller Ω plates despite slightly lower volume fraction [31,34,36,39,44]	Select complete composition–history states; track phase metrics separately	Do not infer optimum chemistry from YS or one cluster snapshot	Matched fracture/FCG across the retained states
Deformation mode and timing	AA2029 YS rises while Ω volume fraction falls; small-stretch APT finds reduced clustering; later warm compression and pre-aging before stretch change the response [58,59,60,61,62,68,73,74]	Record strain definition, local plasticity, starting state and loading time	Cross-paper contrasts do not isolate one causal variable	Same-pedigree mode/timing comparison with thermal-history-matched undeformed controls
Local damage processes	Directional tearing and observed void linkage complement A/B crack paths; thermal exposure lowers J–R-derived Kc with little YS change at 1000 h [15,16,18,102,105]	Sample particles, boundaries and local strain near the tested crack	Related imaging reports are complementary, not independent fatigue tests	Link mapped process history to local damage evolution
Fatigue and fracture endpoints	T6/T6I4 median and mean life rankings differ; strain-controlled cyclic response and aging-dependent long-crack growth supply separate endpoints [40,93,98,99,111]	Keep N_f_, 2N_f_, da/dN–ΔK and J–R measures separate	No pooled curve or strength-to-life conversion	Replication and uncertainty in each relevant crack regime
Manufacturing and exposure	Exposed T3/T8 initiate in different regions despite the same reported fitted stress change; independent AM surfaces reveal lack of fusion [94,95]	Map susceptible regions after the complete route and exposure	AM fracture surfaces do not document weld corrosion	Matched forming × manufacturing/exposure comparisons; intact stress fields
Cross-over design boundary	Cu/Ag strengthening can coincide with reduced corrosion resistance; particle-assisted refinement and paint-bake hardening use distinct routes [46,47,48,49,50]	Compare formable state, hardening route and intended endpoint	Shared solutes do not imply the same phases or damage-tolerance performance	Formed-sheet crack response under common application conditions

**Table 6:** (**A**) Comparison purpose and required controls. (**B**) Specific experimental contrasts.

(**A**)				
**Comparison Purpose**	**Prioritize Matching**	**Permitted Variation**	**Primary Outputs**	**Remaining Mismatch**
Mechanism identification	Material pedigree; local thermal history and downstream treatment; specimen/test conditions as the question permits	Designated strain, load or timing intervention	Affected local state and separately defined damage endpoint; both absolute values	Record residual composition, thermal, geometry and extraction differences; a shared temper label is insufficient
Route optimization	Common application requirements; endpoint definitions, service loading and acceptance conditions	Route-specific forming and aging schedules	Achieved geometry, absolute performance, manufacturing constraints and applicable range	Whole-route differences do not identify a unique microstructural causal mechanism
(**B**)				
**Contrast**		**Matched or Documented Conditions**	**Question Addressed**	
Unformed reference vs. formed location		Material pedigree, final heat treatment and test conditions	Overall effect of the specified forming history	
Thermal-history-matched undeformed control vs. thermo-mechanically formed location		Measured local temperature–time path and downstream aging	Contribution of deformation within the specified thermal path	
Prestrain before aging vs. loading during aging		Chemistry, initial state, exposure and endpoint definitions; stress/strain paths recorded separately	Sequence-dependent microstructure and crack response	
After forming vs. after a manufacturing step		Mapped location and test conditions; geometry and stress redistribution recorded	Retention through removal, joining or surface treatment	
Extracted coupon vs. intact curved element		Local pedigree/microstructure; quantified constraint and residual-stress differences	Limits of transfer to structural response	

## Data Availability

No new data were created or analyzed in this study. Data sharing is not applicable to this article.

## Supplementary Materials

The following supporting information can be downloaded at: https://www.mdpi.com/article/10.3390/ma19204429/s1, Supplementary Methods S1 and Figure S1 document the structured supplementary search and the record-level assessment links. Figures S2 and S3 present pre-aging schedules and forming-route context. Table S1 records selected source-specific conditions; Table S2 separates source-model quantities; Table S3 provides a comparison template; and Table S4 records the seven transfer limits. Reproduced micrographs retain the source-specific credits stated in the captions. The graphical extractions are secondary representations of published data and are not newly generated experimental datasets.

## Nomenclature

**Term/Symbol**	**Meaning**
APT; TEM; STEM; HAADF-STEM	Atom probe tomography; transmission electron microscopy; scanning transmission electron microscopy; high-angle annular dark-field STEM
DFT; EDS; CT; ECDF	Density functional theory; energy-dispersive X-ray spectroscopy; compact-tension specimen; empirical cumulative distribution function
EBSD; IPF; DIC; DVC	Electron backscatter diffraction; inverse pole figure; digital image correlation; digital volume correlation
CAF; HFQ	Creep-age forming; hot forming and quenching
PRDT	Process-retained damage tolerance—the review’s reporting framework
SH; TH	Single-stage homogenization; triple-stage homogenization
YS; UTS; UIE	Yield strength; ultimate tensile strength; unit initiation energy
FCG; LCF; HCF	Fatigue crack growth; low-cycle fatigue; high-cycle fatigue
N_f_; 2N_f_	Cycles to failure; reversals to failure
R; ΔK; da/dN	Minimum/maximum load ratio; stress-intensity range; crack advance per cycle
R_Q_; R_g_	Endpoint retention Q_formed_/Q_control_; FCG-rate ratio g_formed_/g_control_ at matched ΔK, R, and environment
K*; K_res_	Source-model toughness indicator, not measured K_IC_; residual-stress contribution to the mode-I stress-intensity factor
K_IC_; Kc; J–R	Valid plane-strain toughness; source-defined toughness parameter; J-resistance curve
GP; PFZ; KAM	Guinier–Preston; precipitate-free zone; kernel average misorientation (mean local misorientation angle)
RFSSW	Refill friction stir spot welding
Accumulated equivalent inelastic strain	In Figure S3, the cumulative plastic and creep contributions; recoverable elastic strain is excluded
Precipitate descriptors	Number density (per volume or area, as specified); number fraction (number of one type/total counted); volume fraction; dimensions; spatial distribution
IGC; SCC; HAZ; PMZ	Intergranular corrosion; stress-corrosion cracking; heat-affected zone; partially melted zone
RD; L–T; T–L	Rolling direction; loading direction first and crack-growth direction second
AM; LPBF; SEM; BSE; FIB	Additive manufacturing; laser powder bed fusion; scanning electron microscopy; backscattered-electron imaging; focused ion beam
SD; SE; CI; NR; NE; NA	Standard deviation; standard error; confidence interval; not reported; not extracted/resolved; not applicable
