# Supplementary material for: Forming–Aging Pathways and Damage Tolerance in Al–Cu–Mg–Ag Sheets: Mechanisms, Evidence and Implications for Aircraft Skins
Source: Materials (Basel). 2026 Oct 21;19(20):4429. doi: 10.3390/ma19204429 (PMC13706924; doi:10.3390/ma19204429)
Supplement: Supplementary file 1 [file materials-19-04429-s001.zip › materials-4581662-supplementary.pdf]

Supplementary Materials

Forming–Aging Pathways and Damage Tolerance in Al–Cu–Mg–Ag Sheets: Mechanisms, Evidence and Implications for Aircraft Skins

References use the same numerical bibliography as the main manuscript.

Supplementary Methods S1

This article is a critical narrative review, not a comprehensive systematic review. Documented supplementary searches, targeted source assessment and evidence mapping support a question-led synthesis of forming–aging pathways, microstructure and damage-related properties in Al–Cu–Mg–Ag alloys; they do not establish exhaustive coverage of the literature. The author team selected the literature, extracted and interpreted the relevant information, and made the scientific judgments used in the synthesis, drawing on engineering experience in aircraft structures and materials processing. No new, unpublished experimental data are presented; quantitative figures reproduce or descriptively summarize results from the cited literature. No preregistered protocol or independent dual screening was used; these are limits of the procedures reported, rather than criteria that alone determine the review type.

The latest recorded discovery batches were executed in September 2026, principally through ScienceDirect. Exa and SciSpace provided complementary discovery; Zotero supported reference and attachment management. Seven ScienceDirect queries yielded 1003 saved occurrences and 637 distinct Publisher Item Identifiers (PIIs). Of these, 69 matched available full texts: 37 were represented in the accumulated targeted-assessment register and 32 were outside it. Neither an unmatched record nor a record outside that register was automatically excluded. The all-source register contains 110 reports assessed at different depths. The final bibliography contains 126 distinct reports: 82 retained from the original submission and 44 additions. These counts describe different sets; Methods S1 and Figure S1 describe the executed queries and counting relationships.

Selection for a particular synthesis depended on the question the source could answer. The author team prioritized traceable material identity and processing history, a defined observation or endpoint, interpretable test conditions, and a comparison relevant to composition, timing, local damage or structural transfer. Quantitative comparisons required compatible endpoint definitions and sufficient material and test metadata; sources with unresolved decisive conditions were restricted to qualitative use. Reviews and indirect alloy analogues supplied background or methods, while reports retained for a possible later question were designated reserve. Related publications were linked to avoid treating reused baselines as independent evidence. These decisions developed iteratively during the review rather than through a preregistered score. Tables 1–4 and S1 record the conditions that govern use; an internal final-reference record tracks each source's origin, access depth, role and claim location. Measurements, source-author interpretations, model outputs and the present synthesis are distinguished. NR denotes unreported information, NE information not extracted or unresolved, and NA an inapplicable field.

Results measured in Al–Cu–Mg–Ag alloys are identified as target-family evidence, with product form and test conditions retained. Results from other aluminum-alloy systems are used as explicitly identified mechanistic or methodological comparisons, not as direct validation of Al–Cu–Mg–Ag sheets. Evidence obtained from target-family plate, extruded, model-crystal or additively manufactured material is also distinguished from evidence obtained from wrought sheet or formed panels.

Source categories and their evidentiary roles

Source category	Role	Limits
Peer-reviewed journal articles	Primary experimental and analytical evidence for composition, processing, microstructure and measured damage endpoints. Reviews provide context and citation leads.	Full-text methods and source figures/tables govern quantitative use. Journal prestige does not replace assessment of conditions, endpoints or independence.
Technical reports	Historical alloy development, product specifications, test detail and demonstrator evidence, particularly when journal coverage is incomplete.	Identify the issuing organization, report version, product form and test method. Do not assume journal peer review or independent replication.
Conference papers	Development chronology, process demonstrations and supplementary experimental detail.	Link conference and subsequent journal reports of the same program. Cite the version containing the evidence; do not count related reports as independent validation.
Patents	Document proposed process sequences, manufacturing concepts and the limits of process claims.	A patent claim is not independent validation of fatigue, fracture or structural performance. Patents do not provide the principal support for key scientific conclusions.
Preprints	Identify recent or emerging mechanisms with an explicit provisional status.	Flag lack of confirmed journal peer review, verify the version and seek the published counterpart. Preprints are not the principal support for key conclusions.

Record-level counting and access depth

PII means Publisher Item Identifier. The 637-record ScienceDirect cohort was matched to existing inventory records: 69 linked full texts = 37 in the targeted-assessment register + 32 outside that register. These groups are disjoint; the remaining 568 have no linked available full text. Availability outside the register and absent full text are not scientific exclusions. The separate 110-report register includes 60 evidence, 21 background, 19 reserve and 10 scope-exclusion entries. The ten scope decisions used available full-text front matter rather than detailed quantitative extraction. All 110 entries therefore do not represent the same depth of assessment.

The final manuscript bibliography contains 126 distinct cited reports: 82 retained original references and 44 additions. Their source categories are 117 journal publications (including reviews), four technical reports, three conference papers, one patent and one preprint. These are publication counts, not 117 original experiments or 126 independent studies. The final bibliography and the archived 110-report assessment register overlap in 51 reports; 75 cited reports lie outside that register. The remaining 59 register entries are not cited, without treating non-citation as a new exclusion decision. Two of the 44 additions were assessed specifically for the Figure 4 micrograph replacements after the archived search/assessment batches: Lin et al. [45] and Peng et al. [54]. Their full texts, selected panels, experimental conditions and CC BY licenses were checked separately; the historical ScienceDirect and 110-report counts were not revised.

Secondary data, uncertainty and source conflicts

Source tables and identifiable graphical symbols were extracted with explicit locators. For Figure 6b, 54 A and 57 B positions were recovered from the source plot after axis calibration, checked against four independent textual anchors and overlaid on the original. These are graphically extracted coordinates, not counts of independent specimens or an inferred continuous trajectory; unresolved overlaps were omitted and no Paris fit or extrapolation was added. Figure 6c uses all 36 tabulated failures; Figure 7 preserves 19 failures and five run-outs, including the source table/plot disagreement.

For each Figure 6c temper, n = 18 and the empirical cumulative distribution function (ECDF) is $F_n(N) = (1/n)\ \Sigma\ I(N_i \leq N)$, with the sum over i = 1,…,n and I equal to one when the condition is true and zero otherwise. The lifetimes are ordered on the original cycle scale; the sample median is $(N_{(9)} + N_{(10)})/2$ and the arithmetic mean is $(\Sigma\ N_i)/n$. A logarithmic display does not change either calculation. All 36 observations are failures. The five run-outs in the separate 24-observation welded-sheet dataset are marked as censored observations and are not entered as failures in these ECDFs; no pooled lifetime distribution or new S–N fit is calculated.

For Figure 1, graphical error-bar extents are reproduced without assigning an unreported SD/SE/CI definition. Figure 3c reproduces source indentation SD; at least 30 indents on a specimen do not identify an independent mechanical sample size of 30. Graph-reading allowances remain separate from experimental variability. Source inconsistencies are handled individually as detailed below; the selected body-text temperatures remain identified choices, with unresolved differences retained. NR is reserved for unreported fields; NE marks fields not extracted or unresolved in this comparison; NA marks inapplicability. Source-author explanations, model outputs and review proposals are distinguished from measurements.

Software assisted discovery, extraction organization, checking and drafting. The author team determined relevance, comparison membership and interpretation using engineering experience and scientific judgment.

Extraction detail and decisions on source inconsistencies

Temperature discrepancies are recorded for the particular source and state. For the present revision, the selected temperatures follow the source body text, as specified in the authors' figure-revision decisions; the conflicting schematic or caption is retained in the record. This reporting choice does not establish that body text is intrinsically more reliable. Unresolved state assignments or decisive quantitative conflicts restrict the affected comparison rather than being silently corrected.

For Ref. [44], hot rolling is reported as 450 °C in the body and differently in the process schematic; 450 °C is retained for the displayed route, while the solution temperature and duration remain NR. SH/TH elongation error assignments disagree between Section 3.2 and the Conclusions, so elongation error bars are omitted. For Ref. [93], the selected 525 °C solution-treatment temperature follows the body; the discrepancy is retained in the source register. For the final-aging schedule underlying Figure S2, 165 °C likewise follows the body. These choices do not reconcile every discrepancy in those sources.

Ref. [58] describes maximum IGC depths in its text but average depth in the figure description; the displayed 112.1/94.7/86.9 μm values retain the textual maximum-depth definition and source ± values. Conflicting PFZ descriptions are not converted into a monotonic mechanism. Ref. [74] gives different 4 h/6 h peak-aging assignments; its final

microscopy/tensile observations compare complete reported routes without isolating the pre-aging step. Source Fig. 10c–f,h characterizes the state after 100 °C/6 h pre-aging and before stretching: nanoscale Mg/Ag enrichment and image contrast support the authors' co-cluster identification, whereas survival through stretching and vacancy stabilization are mechanistic interpretations. Ref. [39]'s unresolved number-density unit is not used for a numerical density ranking. Ref. [95]'s fatigue sample counts and stress-range/amplitude wording remain unresolved, restricting its use to selected morphology and qualitative interpretation. The Ref. [94] 10,002,000-cycle table entry is retained with its plot disagreement flagged. Ref. [117] gives a 16 h T8 aging duration in the body and 13.6 h in its thermal schematic; no conclusion here depends on selecting one duration.

Figure 1 reproduces graphical error-bar half-spans of approximately 11.1/8.2/5.9/5.9 MPa for YS and 8.4/4.9/7.0/3.1 N/mm for UIE, read from original Figs. 5a and 6a of Ref. [31]. A ±2-pixel reading allowance is recorded separately from experimental variability; no SD, SE or CI definition is inferred. Room-temperature tensile/Kahn crosshead rates were 2/1 mm/min. Figure 2 tensile specimens were parallel to RD, with 20 mm gauge length and 5 mm gauge width.

Figure 3c uses source-text means of 154/163/169/165 HV for compression at 0/20/40/60 min and 162 HV for the uncompressed control; graph-read source SD values are approximately 3.4/2.9/3.9/2.1/2.0 HV. These are indentation scatter, not variability among at least 30 independent mechanical specimens. Figure 5a–c preserves nine source-fitted traces, including small near-zero excursions, with graphical reading allowances of ±0.001 percentage point in strain and ±0.1 h in time. The five AGEFORM markers belong to the 2006 Fig. 43 source and are not renumbered as a figure from the related 2008 report.

Figure 6b retains only directly identifiable published symbols: 54 graphical coordinates for A and 57 for B; these counts do not determine the number of independent specimens. Axis calibration was followed by four independent text-anchor checks and reverse overlays; retained-point records and overlays were kept for the authors' verification. The subset is limited by overlap and cannot be treated as an unbiased reconstruction of the original scatter for mean-curve fitting. Figure 6c uses all 36 tabulated failures, with no run-outs. Figure 7b contains 19 failures and five run-outs. No underlying experiments were added.

For Figure 4, source panels are [31] Fig. 9b,d; [45] Fig. 5b,c; [54] Fig. 2c,f; and [16] Fig. 7a,b. The unaltered IPF key is from the latter Fig. 7a, with vertices 001/101/111 and retained L/T directions. Each image retains its own source, material state and scale bar; adjacency does not imply the same specimen or an independent replication. The image register identifies the editorial confirmation for retaining Figure 4a,b [31], and CC BY 4.0 for Figure 4c,d [45], Figure 4e,f [54], and the reused images from [16, 72, 95]. Lin provides scanning electron microscopy with backscattered-electron contrast (SEM–BSE) images of homogenized residual constituents, whereas Peng provides under-aged/creep-exposed precipitate images. The original quantitative sources [44] and [68] remain the sources of Figures 2 and 3b, respectively; their micrographs are no longer reproduced in Figure 4. In Figure 5e, the original panel letter was removed from its white annotation margin. In Figure 7d,e, a narrow left-edge crop removes the original panel letters; the remaining image pixels and the scale bars are unchanged.

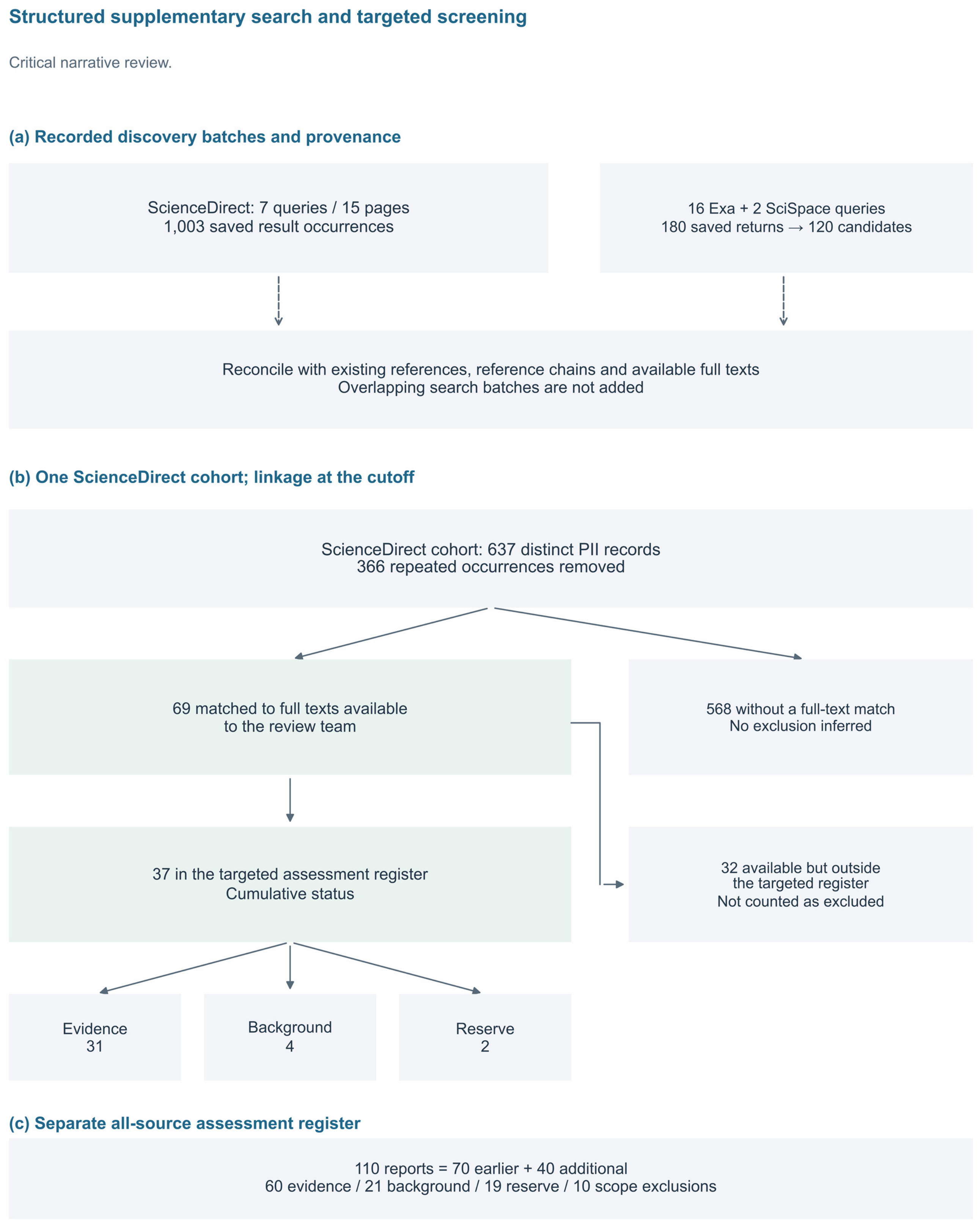

Figure S1. Structured supplementary search and targeted screening workflow in September 2026. ScienceDirect was the primary discovery route; Exa and SciSpace provided complementary discovery. PII means Publisher Item Identifier. The standalone 637-PII node defines the denominator for every solid-arrow branch: 69 matches to available full texts plus 568 without a match; the 69 split into 37 reports in the accumulated targeted-assessment register and 32 outside it. The 37 split into 31 evidence, four background and two reserve reports. These matches include earlier acquisitions/assessments and are not counts of newly downloaded or newly assessed reports in the

latest search batch. Dashed links indicate provenance reconciliation, not additive counts. The separate all-source register contains 110 reports: 60 evidence, 21 background, 19 reserve and ten scope exclusions. The latter were scope checks of available full-text front matter, not ten detailed quality assessments. The 32 available reports outside the targeted register are not classified as excluded. Neither reports nor coordinates are counts of independent studies. The final bibliography is a separate set of 126 cited reports, not the endpoint of the solid-arrow branches. Supplementary Methods S1 describes the record linkage, queries and decision rules. This workflow documents the structured supplementary search and targeted screening adopted for this critical narrative review; it is not presented as a full PRISMA-compliant systematic review. Two reports assessed later for the Figure 4 micrograph replacements enter the final bibliography only; they do not change the archived cohorts shown here.

Executed ScienceDirect search strings

The following strings are preserved as executed, including quotation/escape characters. They are an execution record rather than retrospectively improved searches. The corresponding URLs, collection notes and record-level mappings were retained in the review records. ScienceDirect used the title, abstract or author-specified-keyword field without year, language, journal or article-type filters.

SD-Q01 — ScienceDirect. Saved returns: 223; reported hits: 223; pages: 3.

"\"Al-Cu-Mg-Ag\" OR \"Al-Cu-Ag-Mg\" OR AlCuMgAg OR AlCuAgMg"

SD-Q02a — ScienceDirect. Saved returns: 28; reported hits: 28; pages: 1.

AA2139 OR "AA 2139" OR "2139 alloy" OR "2139 aluminum" OR "2139 aluminium" OR RX226 OR "RX 226"

SD-Q02b — ScienceDirect. Saved returns: 2; reported hits: 2; pages: 1.

AA2029 OR "AA 2029" OR "2029 alloy"

SD-Q03 — ScienceDirect. Saved returns: 237; reported hits: 237; pages: 3.

("Al-Cu-Mg" OR "Al Cu Mg" OR AlCuMg) AND (Ag OR silver)

SD-Q04 — ScienceDirect. Saved returns: 417; reported hits: 417; pages: 5.

(omega OR Ω) AND (aluminum OR aluminium OR "Al-Cu") AND (precipitation OR precipitates OR phase OR platelet)

SD-Q05 — ScienceDirect. Saved returns: 27; reported hits: 27; pages: 1.

("crossover alloy" OR "cross-over alloy") AND (aluminum OR aluminium OR "Al-Mg-Zn")

SD-Q06 — ScienceDirect. Saved returns: 69; reported hits: 69; pages: 1.

AGEFORM OR ("creep age forming" AND (aluminum OR aluminium))

Supplementary Figure S2

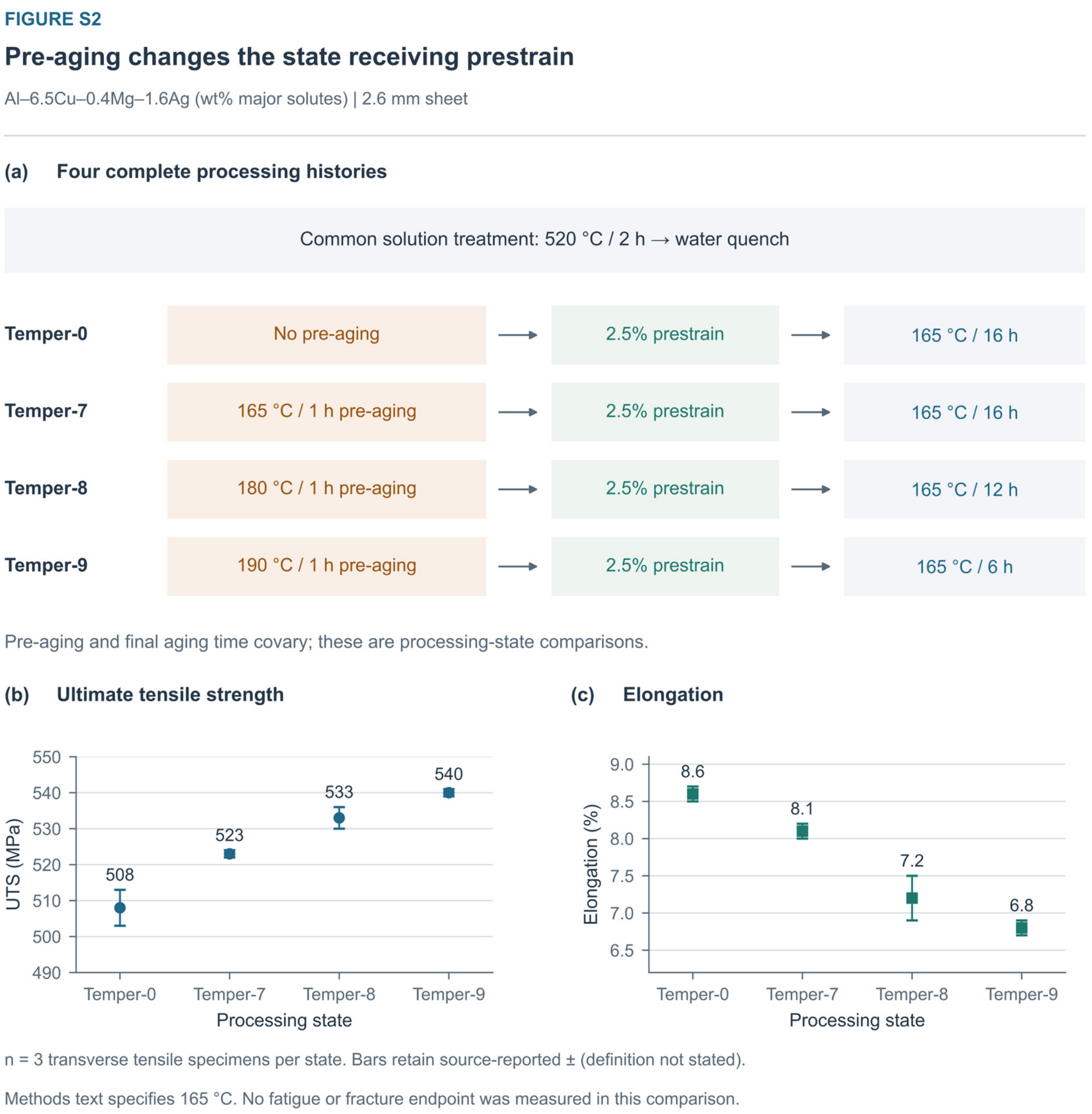

Figure S2. Pre-aging changes the state receiving prestrain in Al–Cu–Mg–Ag sheet [75]. (a) Four source-designated processing states, Temper-0, -7, -8 and -9. The 2.6 mm sheet contained Al-6.5Cu-0.4Mg-1.6Ag-0.3Mn-0.05Ti-0.10Zr (wt%). All states received solution treatment at 520 °C for 2 h and water quenching. Temper-0 received no pre-aging; the other states were pre-aged for 1 h at 165, 180 and 190 °C, respectively. All were then pre-stretched by 2.5% and finally aged at 165 °C for 16, 16, 12 and 6 h. The temperatures follow the source Methods main text and Table 2. Isolated mentions of 160 °C in the pre-aging microstructure discussion are retained as a source inconsistency in the authors' review records and are not substituted for the final-aging recipe. (b) Ultimate tensile strength and (c) elongation, transcribed from the source Table 3. Points are reported averages of three transverse tensile specimens per state; tests were performed at room temperature at 2 mm/min. Bars retain the original ± values, whose statistical definition is not stated; they are not assigned an SD, SE or confidence-interval interpretation. Individual specimen results were not available for replotting. Pre-aging temperature and final-aging duration covary, so the four states do not isolate a pre-aging-temperature effect. Their Ag content differs substantially from AA2139, and these tensile measurements do not establish fatigue or fracture resistance. Numerical data are redrawn; no source micrograph is reproduced.

Supplementary Figure S3

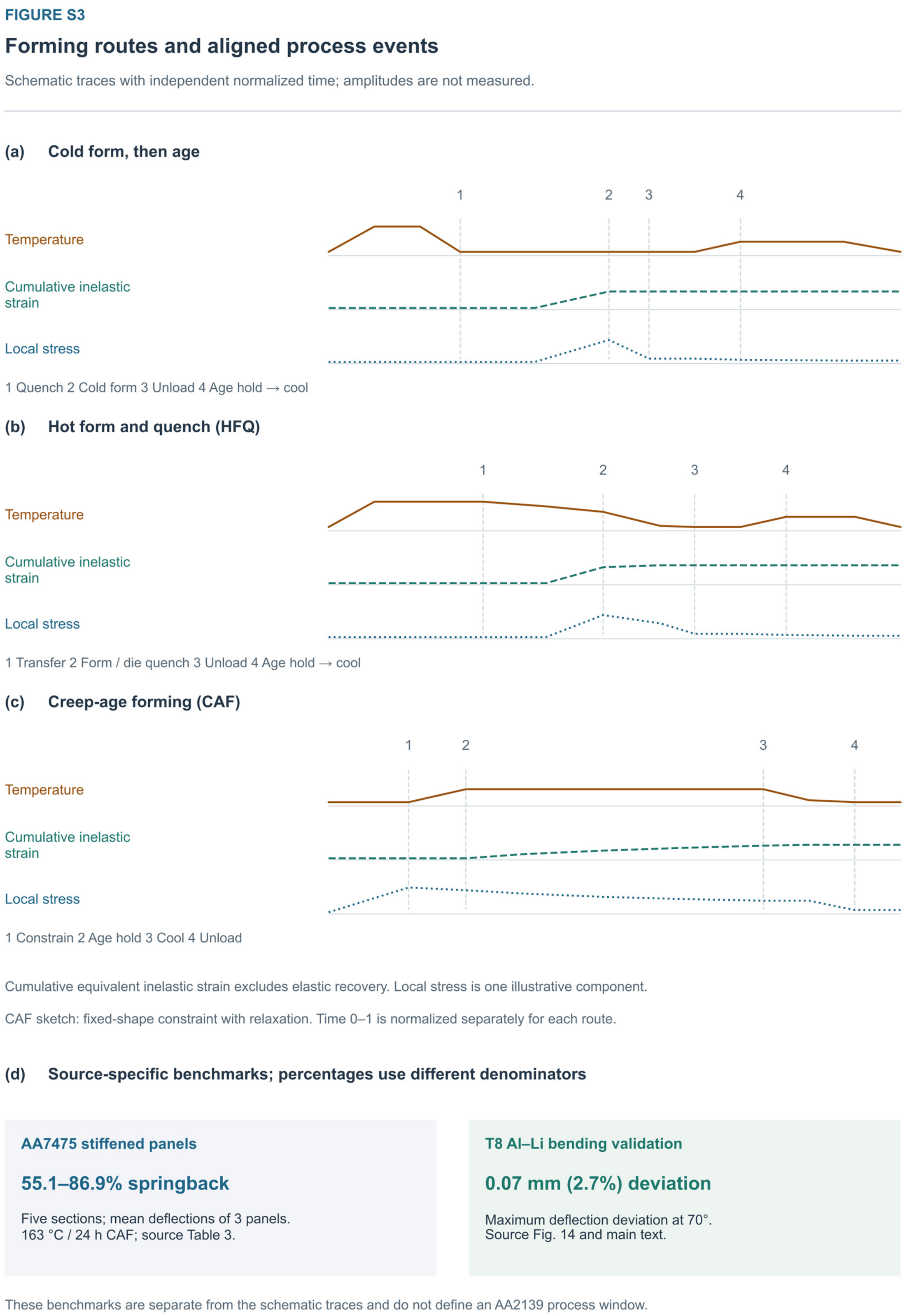

Figure S3. Conceptual forming routes and separately defined creep-age-forming case metrics. (a) Cold forming followed by aging; (b) direct hot form and quench (HFQ); and (c) creep-age forming (CAF). Temperature, accumulated equivalent inelastic strain and one

illustrative local stress component are shown with independent arbitrary vertical scales. Accumulated equivalent inelastic strain includes plastic and creep contributions and does not decrease on unloading; recoverable elastic strain is excluded. Time is independently normalized within each route; neither durations nor curve heights are measured, digitized, simulated or comparable across routes. The CAF sketch illustrates fixed-shape constraint with stress relaxation during the hold; it does not represent every constant-stress aging test. Cooling, unloading and constraint details must be specified for an actual process. The 2024-type patent [86] supports disclosed forming/heat-treatment order only. The prestrain/artificial-aging sequence also has source-specific material context in [75]. The AA6082 study [87] supplies direct-HFQ sequence context and also describes indirect HFQ, in which cold forming of O-temper sheet precedes solution treatment and die calibration/quenching. Its appended erratum corrects the handling editor's name, not experimental results. The study explicitly leaves shape accuracy, including springback, unevaluated. Warm-compression timing experiments are not substituted for HFQ or CAF engineering validation.

Numbered guides align source-independent process milestones with the three schematic traces; they do not quantify physical process durations. (d) Two distinct quantitative contexts. In [90], three AA7475 stiffened panels, 600 × 300 mm with variable local thickness, were machined from 50.8 mm parent plate after 480 °C/3 h solution treatment, water quenching and 121 °C/6 h pre-aging. CAF was performed at 163 °C for 24 h under 7 atm. The five section springback values in Table 3, derived from mean deflections of the three panels, are 86.9, 75.4, 65.5, 59.2 and 55.1%; the displayed 55.1-86.9% range describes section variation. The source table states "±1% max." without identifying an SD or confidence interval; that statement is not used as the meaning of the across-section range. In [91], the maximum reported simulated-minus-experimental deflection was 0.07 mm at 70° to the rolling direction, or 2.7% using 100 × (simulation - experiment)/experiment. The case used T8 third-generation Al–Li material (0.6-0.9 wt% Li), 220 × 18 × 2 mm four-point-bending specimens, 20.2 mm loading displacement and 5 h CAF. Experimental validation points in the source Figure 14 are attributed there to Wang et al. (2023b); they are not newly independent repetitions in the 2024 modeling paper. The 2.7% is a prediction deviation, not springback or a statistical error interval. These sources define neither a common AA2139 operating window nor a pooled springback distribution.

Table S1. Selected source-specific conditions

This selected-source register complements the main comparative tables. The table identifies the conditions that constrain each selected comparison; detailed claim-to-source and version records were retained during preparation. It is not a count of all eligible literature or independent experiments. WQ denotes water quenching.

Source / state	Product / specimen	Cu/Mg/Ag (wt%)	Initial heat / processing history	Plotted intervention	Evidence used	Interpretation boundary
[31] 0.6 Mg	1.6 mm sheet	5.02/0.61/0.41	360 °C/6 h → 470 °C/6 h → 520 °C/24 h; solution 520 °C/1 h; WQ	2% pre-stretch; 160 °C/14 h	TEM and measured YS/UIE; model K* and strengthening share separately in Table S2	Chemistry and thermal history covary; no pure Mg effect
[31] 0.8 Mg	1.6 mm sheet	5.08/0.83/0.42	360 °C/6 h → 470 °C/6 h → 515 °C/24 h; solution 515 °C/1 h; WQ	2% pre-stretch; 160 °C/18 h	TEM and measured YS/UIE; model K* and strengthening share separately in Table S2	Chemistry and thermal history covary; no pure Mg effect

Source / state	Product / specimen	Cu/Mg/Ag (wt%)	Initial heat / processing history	Plotted intervention	Evidence used	Interpretation boundary
[31] 1.0 Mg	1.6 mm sheet	5.05/0.97/0.41	360 °C/6 h → 470 °C/6 h → 510 °C/24 h; solution 510 °C/1 h; WQ	2% pre-stretch; 160 °C/22 h	TEM and measured YS/UIE; model K* and strengthening share separately in Table S2	Chemistry and thermal history covary; no pure Mg effect
[31] 1.5 Mg	1.6 mm sheet	5.04/1.52/0.44	360 °C/6 h → 470 °C/6 h → 500 °C/24 h; solution 500 °C/1 h; WQ	2% pre-stretch; 160 °C/32 h	TEM and measured YS/UIE; model K* and strengthening share separately in Table S2	Chemistry and thermal history covary; no pure Mg effect
[44] SH/TH	2 mm sheet	5.02/0.61/0.41	SH 520 °C/24 h vs TH 360 °C/6 h → 470 °C/6 h → 520 °C/24 h; rolling 450 °C; anneal 420 °C/30 min; solution details NR; WQ	2% pre-stretch; 160 °C/14 h	Ω descriptors and tensile properties in Figure 2; source TEM interpretation discussed in text	Shared central values with [31] are not independent replication; source micrographs are not reproduced in Figure 4
[58] AA2029	3 mm sheet	3.62/0.90/0.45	513 °C/1.5 h; WQ	1.8/2.5/3.4% tension → 175 °C peak aging ≈17/16/16 h	TEM-derived Ω, tension and maximum IGC depth	Cluster mechanism is source interpretation, not APT
[68] bicrystal	Bicrystal; grain A/B and near-boundary regions	1.96/0.24/0.18	525 °C/2 h; WQ	0/50/100 MPa during 180 °C/53 h; yielding at 100 MPa	Regional Ω volume fraction in Figure 3b; source micrographs are not reproduced	Grain-interior and near-boundary categories are distinct; not the material or comparison shown in Figure 4e,f

Source / state	Product / specimen	Cu/Mg/Ag (wt%)	Initial heat / processing history	Plotted intervention	Evidence used	Interpretation boundary
[73] timing probe	Φ3 × 10 mm cylinder machined from 3 mm sheet	5.13/0.97/0.38	515 °C/2 h; natural aging four days	5% compression at 0.01 s^{-1}, applied at 0/20/40/60 min during a 200 °C/60 min hold; separate undeformed control	Hardness means and indentation SD; source Fig.2b	Warm compression timing, not tensile prestrain or whole-sheet CAF
[16] A/B	5 mm L–T CT plate specimens	4.00/1.45/0.54	A 320 °C/4 h; B 420 °C/4 h; both 490 °C/20 min, WQ, natural aging (duration NR)	Long-crack growth: R=0.1, 10 Hz, room air	EBSD crack-path maps and FCG from the same source study	Not AA2139; no specimen-by-specimen pairing inferred
[45] P2/P3	Industrial ingot; bottom D/4 region	5.23/0.94/0.62; Mn 0.66; Zr 0.12	420 °C/5 h → 480 °C/8 h	510 °C/24 h (P2) or 515 °C/24 h (P3); WQ	Figure 4c,d: SEM–BSE residual constituents; source Fig. 5b,c; bars 500 µm; CC BY 4.0	Homogenized ingot, not peak-aged sheet or a damage endpoint; no area-fraction values extracted from source Fig. 5f
[54] under-aged / post-creep	Extruded bar; creep gauge 4 × 3 mm, length 20 mm	6.00/0.40/0.40; Mn 0.30; Ti 0.10; Zr 0.20; Sc 0.10	500 °C/24 h; extrusion 430 °C, 7:1; solution 515 °C/2 h; WQ; under-aging 180 °C/8 h	Under-aged state versus subsequent creep at 200 °C/250 MPa/50 h	Figure 4e,f: near-<110>Al bright-field TEM; source Fig. 2c,f; bars 100 nm; CC BY 4.0	Additional heat and stress act together; not stress-only, not AA2139 sheet, not CAF or same-field imaging

Additional comparison records for the expanded synthesis:

Reference	Material / contrast	Thermal sequence	Endpoint and boundary
[38]	Al–5.1Cu–1.0Mg with 0 or 0.4 wt% Ag; within-study Ag contrast	185 °C; 11 h peak and later 18/100 h states	Tensile retention and Ω/S morphology; no fatigue or K_{IC} test; not designated AA2139

Reference	Material / contrast	Thermal sequence	Endpoint and boundary
[59]	AA2139 plate; nominal 7% cold stretching versus rolling	Source-specific deformation and aging histories	Engineering stress–strain curve area, called “toughness” in source Section 3.4/Fig. 14 (PDF pp.9–10); relative change reported. Explicit integration limits/absolute energy units NR. Qualitative tensile work metric only; related lineage to [60]
[60]	Industrial AA2139 plate; 5–15% cold rolling versus undeformed state	Solution/quench, deformation, storage and aging	Source Section 3.3.3/Eq. (2), Fig. 13c/Table 4 (PDF pp.10–11): $W(\varepsilon) = \int_0^{\varepsilon} S\,de$, engineering stress S and strain e. Integrated to the reported tensile endpoint; MJ/m^3 denotes work per initial volume, not mass-specific absorption or fracture energy per area
[18]	Al–Cu–Mg–Ag, 2.3 mm sheet; L–T CT, W = 50.8 mm	T8; 135 °C exposure for 0/1000/3000 h	J–R-derived Kc at Δa = 2 mm; 90/74/75 MPa√m; no resolved 74→75 recovery without uncertainty; fracture n NR
[98]	Al–4.5Cu–0.56Mg–0.77Ag; ≈20 mm parent plate; round gauge Φ5 × 18 mm, L direction	T6 vs T840 (40% rolling); aging 190 °C/2 h	Fully reversed constant total strain amplitude 0.4–1.0%, 0.5 Hz; $2N_f$ convention; fatigue n per amplitude NR; tensile ≥3 is separate
[99]	Same alloy family; ≈20 mm parent plate; round gauge Φ5 × 12.5 mm, longitudinal	520 °C/1 h → WQ; T83 3% stretch → 190 °C/2 h	Symmetric constant total strain amplitude 0.4–1.5%, 0.5 Hz; independent fatigue n NR; T6/T840 comparison lineage linked to [96]
[40]	Cu 3.19/3.51/3.78 wt%; 50 × 48 × 2.5 mm fatigue specimens (Fig. 1c)	Separate peak-aged states. Precracking: approximately 2 mm at Kmax = 10 MPa√m	FCG: source-reported cyclic load-control program; R, frequency and independent n NR. Source “constant load” wording does not specify Pmax/Pmin or constant ΔK; separate IGC
[46]	Al–Mg–Zn with Cu/Ag additions; single- versus two-stage aging	Source-specific low-temperature precipitation routes	Hardness, tension and microscopy; not Ω-alloy fatigue evidence
[47]	Al–Mg–Zn–(Cu,Ag) cross-over alloys; single- versus two-stage overaging	125 °C/45 days versus 100 °C/3 h + 175 °C/16 days	Strength and corrosion under the examined conditions; not a universal corrosion ranking or AA2139 evidence
[48]	Cross-over alloy clustering; prolonged low-temperature aging	60 °C/42 days	APT, TEM and tensile response; not a short paint-bake treatment or a damage-tolerance test

Reference	Material / contrast	Thermal sequence	Endpoint and boundary
[49]	1 mm industrial Al–4.91Mg–3.46Zn–0.46Cu sheet; 58%/85% cold reduction variants	Distinct routes: 380 °C/5 min recrystallization; 480 °C/1 min solution anneal; R5 adds ≈24 h at 470 °C after solution/WQ	Separate hardenability sequence: 480 °C/1 min → water/air quench → 100 °C/5 h → room temperature 14 d → 185 °C/20 min; no single inferred forming/solution sequence
[50]	Automotive Al–Mg–Zn–Cu sheet; pre-aging, paint bake and forming	Source-specific low-temperature pre-aging and paint bake	Forming limits and bake hardening; GP/η′ evidence does not establish T-phase identity
[74]	2 mm Al–5.51Cu–0.61Mg–0.62Ag sheet; direct 5% stretch vs pre-aged then 5% stretch	Both 525 °C/1 h → WQ; PIA: 5% stretch → 165 °C aging. P-PIA: 100 °C/6 h → 5% stretch → 165 °C aging. Source Fig. 1 assigns 6 h, while 4 h/6 h peak descriptions conflict	Fig. 10c–f,h (PDF pp.10–11) observes the pre-aged, pre-stretch state: contrast and local Mg/Ag enrichment identified by the authors as co-clusters. DFT concerns vacancy-related interpretation. Final TEM/tensile results compare complete routes, not an isolated pre-aging effect; no fatigue or K_{IC} test
[101]	AA2519 + Mg + Ag (3.2 mm) and C416 (2.3 mm); L–T CT, W = 76.2 mm	T87 and T82 source states; J-integral tests at ambient/elevated temperatures	Full-text methods and results consulted; initiation and plane-stress growth resistance remain separate
[106]	AA2519 + Mg + Ag, 3.2 mm hot-rolled T87 sheet	529 °C/1 h → WQ → 7% stretch → 177 °C/3 h	Void-sheet surfaces at 25/150/175 °C; notched bars at 25/150 °C; not AA2139
[111]	Al–4.1Cu–1.45Mg–0.54Ag; 2 mm sheet; L–T CT 47.5 × 38 × 2 mm	495 °C/1 h → WQ; T351 (2% pre-stretch) vs 170 °C/0.5 h; subsequent 150 °C exposures	FCG at R = 0.1, 10 Hz, room air; source targets rates above 10^{-5} mm/cycle; independent n NR
[117]	Two Al–Cu–Mg–Ag alloys; ≈2.25 mm parent sheet, ≈1.6 mm stressed coupons	T8/T8+; T8+ adds 5000 h at 107.2 °C; T8 aging time differs between body and diagram	Alternate immersion: 0.6 M NaCl, 10 min wet/50 min dry; three-point stressed and unstressed comparisons; corrosion/SCC, not cyclic fatigue
[122]	AA7050-T7451 machined pockets	Initial residual stress and toolpath comparison	Qualitative distortion–initial-stress–toolpath relationship

Table S2. Source-model quantities kept separate from measured endpoints

Nominal Mg (wt%)	Model K* (MPa√m)	Ω share of modeled precipitation strengthening (%)	Source
0.6	42.5	87.0	[31], Table 4 and Section 4.1
0.8	51.3	85.8	[31], Table 4 and Section 4.1
1.0	55.1	83.5	[31], Table 4 and Section 4.1
1.5	32.2	68.3	[31], Table 4 and Section 4.1

K* denotes the source-model toughness indicator, not measured plane-strain K_{IC}. Ω strengthening share is not phase volume fraction. Source-specific experimental conditions are listed in Table S1.

Table S3. Template for recording a matched comparison

Intervention / control	Material and location	Matched endpoint / test	Absolute control	Absolute intervention	Ratio and meaning	Mismatch / missing data
720 h vs 0 h salt-spray pre-exposure [94]	3.2 mm AA2139 laser-welded sheet; T3/T8 before welding	Author-fitted σ_{max} at 10^7 cycles; R = 0.1, 30 Hz	115 MPa	55 MPa	55/115 = 0.4783; exposure stress ratio	Forming-retention test: NA; independent fit uncertainty: NR; no new S–N fit
[enter intervention and reference]	[enter pedigree, form, thickness, mapped location]	[enter endpoint, geometry, orientation, loading, environment]	[value + unit + uncertainty + n]	[value + unit + uncertainty + n]	[defined numerator/denominator; favorable direction]	[remaining mismatch; distinguish NR / NE / NA]

Editable reporting template; the example is fatigue after salt-spray pre-exposure, not validation of forming retention. Report absolute values alongside ratios. For g = da/dN compare at matched ΔK, R and environment; a lower g-ratio is favorable. No universal pass ratio or weighted damage-tolerance score is proposed.

Table S4. Limits of transferring coupon evidence to a curved structure

Variable	Why it affects transfer	How to handle it
Curvature	Changes loading and local constraint	Map local curvature and validate representative curved elements
Thickness	Changes through-thickness gradients and crack constraint	Retain parent, specimen and local final thickness separately
Constraint	Coupon and reinforced structure impose different multiaxial states	Specify geometry/restraint; use validated local fields
Residual stress	Extraction and processing can redistribute the crack-driving field	Measure or reconstruct fields before/after extraction with uncertainty
Crack geometry	Initiation site, aspect ratio and crack size alter driving force	Specify actual defects/precracks and verify relevant crack regimes
Boundary conditions	Load introduction and restraint alter structural response	Reproduce service-relevant load and support conditions
Manufacturing sequence	Joining, removal and surface treatment change local state	Track location through the actual order of operations; pair endpoint comparisons

Review synthesis informed by [8, 15, 16, 72, 94, 126–128]. Related EMST reports [126–128] are one program lineage, not three independent demonstrations of AA2139 processing retention.
